# Giant single-step upconversion via sub–35-fs phonon dynamics in the nonlinear optical regime

**DOI:** 10.1126/sciadv.adx1686

**Published:** 2025-10-15

**Authors:** Jingjing Yao, Yahui Li, Xiaguang Zhang, Heyuan Liu, Ming Xia, Chong Hu, Qiu Wang, Jun Yi, Jeongmin Kim, Hailong Chen, Enzheng Shi, Xiaoze Liu

**Affiliations:** ^1^ School of Physics and Technology, Center for Nanoscience and Nanotechnology, and Key Laboratory of Artificial Micro- and Nanostructures of Ministry of Education, Wuhan University, Wuhan 430072, China.; ^2^ Wuhan University Shenzhen Research Institute, Shenzhen 518057, China.; ^3^ Research Center for Industries of the Future and School of Engineering, Westlake University, Hangzhou 310030, China.; ^4^ Key Laboratory of Green Chemical Media and Reactions, Ministry of Education, Collaborative Innovation Center of Henan Province for Green Manufacturing of Fine Chemicals, College of Chemistry and Chemical Engineering, Henan Normal University, Xinxiang 453007, China.; ^5^ Laboratory of Soft Matter Physics, Beijing National Laboratory for Condensed Matter Physics, Institute of Physics, Chinese Academy of Science, Beijing 100190, China.; ^6^ School of Physical Science, University of the Chinese Academy of Sciences, Beijing 100049, China.; ^7^ Songshan Lake Materials Laboratory, Dongguan 523808, Guangdong, China.; ^8^ School of Information Technology, Wuhan University of Technology, Wuhan 430070, China.; ^9^ State Key Laboratory of Physical Chemistry of Solid Surfaces College of Chemistry and Chemical Engineering School of Electronic Science and Engineering, Fujian Key Laboratory of Ultrafast Laser Technology and Applications, Xiamen University, Xiamen 361005, China.; ^10^ Innovation Laboratory for Sciences and Technologies of Energy Materials of Fujian Province (IKKEM), Xiamen 361005, China.; ^11^ Department of Applied Bioengineering, Graduate School of Convergence Science and Technology, Seoul National University, Seoul 08826, Republic of Korea.; ^12^ Research Institute for Convergence Science, Seoul National University, Seoul 08826, Republic of Korea.; ^13^ Wuhan Institute of Quantum Technology, Wuhan 430206, China.; ^14^ Wuhan National High Magnetic Field Center, Huazhong University of Science & Technology, Wuhan 430074, China.

## Abstract

Phonon-assisted upconversion (UC) for anti-Stokes photoluminescence stands as a fundamental and widely studied process, central to both ultrafast electron-phonon coupling physics and diverse photonic applications. However, the ultrafast dynamics limit of UC has yet to be addressed, preventing its integration into the nonlinear optical regime. Here, we find a giant single-step UC of ~200 milli–electron volts via sub–35-femtosecond phonon dynamics in two-dimensional hybrid organic-inorganic perovskites in the nonlinear regime. The single-step UC approaches the phonon dynamics limit of ~23 femtoseconds and gains energy about eight times the room-temperature thermal energy (~25 milli–electron volts), enabling its synergistic integration into the nonlinear regime. Benefiting from the unique electron-phonon coupling between organic vibrations and excitons in inorganic lattices, the UC demonstrates distinctive signatures of Raman anisotropy and strong nonlinearity. This work opens new avenues for studying uncharted phonon dynamics and nonlinear optical mechanisms, offering substantial advantages in optical refrigeration, upconverting energy harvesting and optical microscopy.

## Teaser

Ultrafast coupling between organic vibrations and inorganic excitons enables giant upconversion in the nonlinear optical regime.

## INTRODUCTION

Upconversion (UC), the process of emitting photons with higher energy than the excitation photons and known as anti-Stokes photoluminescence (ASPL), has become one fundamental optical phenomenon in solids (*1*–*3*). In the linear regime, UC energy gain arises from electron-phonon coupling, i.e., phonon-assisted UC (*4*). This process can be substantially enhanced by the resonances of excited states (*5*) and fundamentally modulated by structural properties and phonon dynamics (*6*). With these properties, the phonon-assisted UC enables applications such as macroscopic optical cooling, upconverting energy harvesting, and enhanced optical microscopy (*3*, *5*, *7*–*12*). In the nonlinear regime, UC energy gain results from two-photon (TP) absorption (TPA), which involves either virtual or excited states (e.g., excited states of rare-earth atomic orbitals or organic triplet states) and typically occurs on ultrafast timescales (*13*–*24*). TPA not only depends on the nonlinear dielectric properties of materials but also demonstrates intriguing nonlinear effects by engineering resonance effects of excited states (*25*). By leveraging the nonlinear effects, TPA facilitates energy harvesting (*23*, *26*–*30*) and optical microscopy (*22*, *31*, *32*). Despite extensive research into ASPL and the growing recognition of electron-phonon coupling’s role in nonlinear optics (*31*, *33*–*38*), phonon-assisted UC and TPA have been traditionally studied as distinct and independent processes.

The synergy of phonon-assisted UC and TPA could offer a fresh perspective to explore nonlinear optical physics with electron-phonon coupling and diverse applications with improved thermal management, desired nonlinear effects, and extended spectral ranges (*24*, *30*, *35*). However, phonon-assisted UC has been hardly observed in the nonlinear optical regime in solids, primarily due to its mismatched dynamics and fundamentally distinct mechanisms from TPA. To realize the synergy of phonon-assisted UC and TPA with compatible mechanisms and aligned ultrafast dynamics, it requires a material with several stringent criteria: sufficiently high photoluminescence (PL) quantum yield, efficient electron-phonon coupling, and strong optical nonlinearity. The first criterion ensures the experimental detection of both phonon-assisted UC and TPA, while the latter two promise the coexistence of both processes and their effective synergy with aligned dynamics in a single material. It is nevertheless challenging to meet all these criteria in conventional UC materials, such as rare-earth doped materials (*19*–*22*, *39*), organic semiconductors, and low-dimensional materials (*5*, *7*, *9*–*11*).

In this work, we report the observation of a giant single-step UC of ~200 meV via sub–35-fs phonon dynamics in two-dimensional perovskites (2DPKs) in the nonlinear optical regime. The single-step UC approaches the phonon dynamics limit of ~23 fs and gains energy (~200 meV) about eight times the room-temperature thermal energy (~25 meV), enabling its synergistic integration into the nonlinear regime. Typical single-step UC in other material systems ranges from 10 to 165 meV (table S1), marking the observed energy gains of ~200 meV as giant single-step UC. With these highlighted features, the UC is observed by a combination of spectroscopic measurements, material characterizations, and density functional theory (DFT) calculations. Because of its ultrafast dynamics limit, the phonon-assisted UC may be aligned with TPA, resulting in synergistic TPA and ASPL, namely TP-ASPL in the nonlinear regime. This synergy is achievable because its criteria can be all satisfied in a specific group of hybrid organic-inorganic 2DPKs with 2T^+^ (bithiophenylethylammonium) cations. Specifically, the inorganic octahedral lattice ensures high PL quantum yield and efficiently enables strong optical nonlinearity via 2D exciton resonances (*25*). Meanwhile, the organic 2T^+^ cations in the outer layer facilitate sub–35-fs electron-phonon coupling via dominant high-frequency localized vibrational modes (fig. S3), in contrast to previously reported UC via collective phonons in the inorganic lattices (*5*, *6*). The unique coupling between organic vibrations and excitons in inorganic lattices gives rise to distinctive signatures of UC anisotropy and strong nonlinearity, offering substantial advantages for diverse photonic applications in optical cooling, near-infrared optoelectronics, and optical microscopy.

## RESULTS

### Observation of phonon resonances, ASPL, and TP-ASPL

**Fig. 1.**
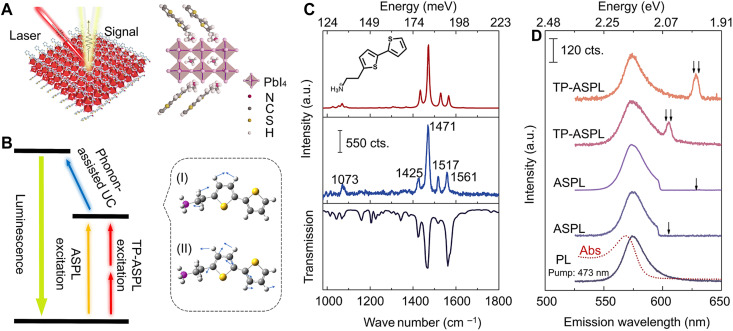
Schematics and characterizations of ASPL and TP-ASPL in (2T)_2_(MA)Pb_2_I_7_. (**A**) Schematics of experimental configuration for ASPL and TP-ASPL in (2T)_2_(MA)Pb_2_I_7_. The zoomed-in schematic on the right represents the (2T)_2_(MA)Pb_2_I_7_ crystalline structure. (**B**) Schematics of the ASPL and TP-ASPL mechanisms. ASPL (TP-ASPL) could occur by one-photon (TP) excitation with phonon-assisted UC by the 2T^+^ cations. (I) and (II) represent the zoomed-in 2T^+^ with two typical vibrations of resonances at 1073 and 1561 cm^−1^, respectively. (**C**) Phonon characterizations of (2T)_2_(MA)Pb_2_I_7_. Top panel: DFT calculations of the vibration modes in 2T^+^; middle panel: Raman spectrum excited at 633 nm; bottom panel: FTIR spectrum. These phonon characterizations are consistent from calculations to experiments. a.u., arbitrary units. (**D**) PL, ASPL, and TP-ASPL spectra of (2T)_2_(MA)Pb_2_I_7_. The red dashed curve represents the absorption spectrum with a minimal Stokes shift of ~5 nm. All the spectra peak at 575 nm with similar profiles, while there are also SHG peaks in the TP-ASPL spectra. The excitations of ASPL (at 612 and 633 nm) and TP-ASPL (at 1228 and 1270 nm) are indicated by the arrows.

The experiments were performed in a typical 2DPK material, i.e., (2T)_2_(MA)Pb_2_I_7_ in the general formula of (2T)_2_(MA)_*m*−1_Pb*_m_*I_3*m*+1_, where MA^+^ represents methylammonium and the layer thickness integer *m* is 2 (Fig. 1A). Here, (2T)_2_(MA)Pb_2_I_7_ (see Materials and Methods, fig. S1, and table S2 for material details) is studied for better demonstration, while (2T)_2_PbI_4_ (layer integer *m* = 1) could also show similar phenomena and would be discussed later. In this experimental configuration, the energy diagram sketches the ASPL mechanism of one-photon/TP excitation with phonon-assisted UC (Fig. 1B). The phonons participating in UC only come from the organic cations, as illustrated by two typical vibrations in the zoomed-in schematics of 2T^+^. These vibrations can be simulated by DFT calculations with the 2T^+^ structure (Materials and Methods and fig. S2) and directly observed by Raman spectroscopy and Fourier transform infrared spectroscopy (FTIR) (Materials and Methods). In Fig. 1C, typical Raman modes are identified with five peaks at 1073 cm^−1^ (133 meV), 1425 cm^−1^ (177 meV), 1471 cm^−1^ (182 meV), 1517 cm^−1^ (188 meV), and 1561 cm^−1^ (194 meV), which are in excellent agreement with the FTIR spectrum and DFT calculations. The vibrational resonances of these high-frequency modes are independent from the collective phonons of the inorganic lattice, which can be further confirmed by the calculated phonon density of states and low-frequency Raman spectroscopy (fig. S3). These vibrational resonances are critical to the luminescence spectra of Fig. 1D, which include PL excited at 473 nm, ASPL (one-photon excitation) excited at 612 and 633 nm, and TP-ASPL excited at 1228 and 1270 nm. All of them share the same peak at 575 nm, corresponding to the quantum-confined 2D exciton in 2DPKs (*40*, *41*). For the ASPL and TP-ASPL spectra, the single arrow represents the excitation energies, while the double arrows represent twice the excitation energies. In both TP-ASPL spectra, there are second-harmonic generation (SHG) peaks with broken inversion symmetry (see details in fig. S4) (*42*). ASPL (TP-ASPL) spectra show that the luminescence photons acquire giant energy gains than (twice) the excitation photons. These giant gains are close to the phonon resonances at 1073 cm^−1^ (133 meV) and 1561 cm^−1^ (194 meV).

### Spectroscopic investigation of single-step phonon-assisted UC

**Fig. 2.**
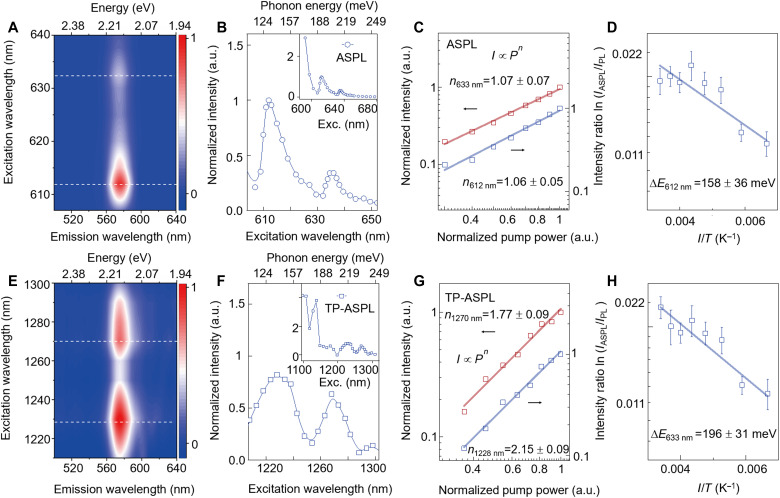
PLE, power dependences, and temperature dependences of ASPL and TP-ASPL. (**A** and **E**) Color maps of ASPL spectra (A) and TP-ASPL spectra (E) as a function of excitation wavelengths. The color bars represent the normalized intensities. The dashed lines mark the ASPL peaks at 612 and 633 nm (A) and TP-ASPL peaks at 1228 and 1270 nm (E). (**B** and **F**) Excitation spectra for ASPL (B) and TP-ASPL (F), where the detection is parked at the PL peak of 575 nm. In (B), the excitation is scanned from 590 to 675 nm (full range in the inset) with a fixed power of 0.4 μW. In (F), the excitation is scanned from 1098 to 1308 nm (full range in the inset) with a fixed power of 1 mW. (**C** and **G**) Pump power dependences of ASPL (C) and TP-ASPL (G). The power dependences of ASPL excited at 612 and 633 nm show power indices of ~1 for one-photon excitation, while those of TP-ASPL excited at 1228 and 1270 nm show power indices of ~2 for TP excitation. (**D** and **H**) Intensity ratio versus inverse temperature with excitation at 612 nm (D) and excitation at 633 nm (H). The fitted slopes represent the activation energies, which are consistent with the phonon energies. Note that all the normalized intensities and intensity ratios in [(C), (D), (G), and (H)] are based on spectrally integrated intensities.

To clarify the phonon-assisted UC, PL excitation (PLE) spectroscopy was carried out for both ASPL and TP-ASPL. In the PLE spectrum with wavelengths longer than the excitonic peak of 575 nm (Fig. 2, A and B), two resonance peaks can be seen around 612 and 633 nm. By comparing these PLE resonances with the exciton peak, two energy gains of (130 ± 17) and (197 ± 16) meV can be obtained when the linewidths of the excitation laser (~10 nm) are considered. These gains are in resonance with the phonon energies: The energy gain of (130 ± 17) meV is resonant with the 1073 cm^−1^ phonon (133 meV); the energy gain of (197 ± 16) meV is resonant with 1471 cm^−1^ (182 meV), 1517 cm^−1^ (188 meV), and 1561 cm^−1^ (194 meV) phonons. These resonances strongly suggest that ASPL is enabled by a single-step UC of one-phonon absorbance upon excitation at 612/633 nm. Other two resonance peaks around 1228 and 1270 nm can be also resolved as the PLE spectrum extends to the TP excitation range (Fig. 2, E and F). Their TP energies are consistent with PLE peaks of ASPL, suggesting a unique TP-ASPL in the nonlinear regime where the synergy of single-step phonon-assisted UC and TPA is achieved.

To elucidate the phonons’ role, power-dependent and temperature-dependent spectroscopy was performed. In the power dependences of ASPL (TP-ASPL) (Fig. 2, C and G), the power law of $I\propto P^{n}$ (where *I* is the PL intensity, and *P* is the pump power) shows power indices *n* of ~1 (~2), confirming one-photon (TP) excitation. These power dependences indicate that giant UC results from only the phonons without the involvement of extra photons. Temperature dependences of the intensity ratio of ASPL to PL ( $I_{\text{ASPL}}/I_{\text{PL}}$ ) could provide critical evidence to clarify the phonons’ role (Fig. 2, D and H, and fig. S5). The ratio ( $I_{\text{ASPL}}\;/\;I_{\text{PL}}$ ), representing the strength of electron-phonon coupling in the UC, decreases with temperature (*T*) and can be modeled with as $I_{\text{ASPL}}\;/\;I_{\text{PL}}\propto \text{exp}\left(-\frac{∆E}{k_{B}T}\right)$ (*2*, *7*, *43*), which follows an Arrhenius trend by approximating the phonon occupation number (note S1). Here, $k_{B}$ is the Boltzmann constant, and ∆E represents the fitted activation energy, which refers to the phonon resonances in this work. For the excitation at 612 nm (633 nm), the fitted ∆E is (158 ± 36) meV [(196 ± 31) meV]. The fitted ∆E of (158 ± 36) meV is resonant with the 1073 cm^−1^ phonon (133 meV); the other fitted ∆E of (196 ± 31) meV is resonant with the other four phonons around 1500 cm^−1^ (~ 190 meV; Fig. 1C). The resonant ∆E with phonons indicates that the UC occurs by a single step of one-phonon absorbance through the coupling between the organic vibrations and excitons in inorganic lattices.

Notably, this UC energy gain, up to ~200 meV, is about eight times the room-temperature thermal energy and marks one of the largest phonon-assisted UC (*3*, *6*, *8*). Slightly away from the one-phonon resonance, ASPL could occur even at the excitation of 650 nm with an energy gain of up to ~250 meV (fig. S6), setting the upper limit for this giant UC. To further verify the UC mechanism, we have studied a similar UC process in the 2D perovskite of (3T)_2_(MA)Pb_2_I_7_, where the inorganic lattices have similar quantum well excitonic features, while the organic cations [3T^+^: 2-([2,2′:5′,2″-terthiophen]-5-yl)ethan-1-aminium] provide different vibrational modes from (2T)_2_(MA)Pb_2_I_7_ (fig. S7). The UC energy gains in (3T)_2_(MA)Pb_2_I_7_ exhibit systematic shifts via the vibrational modes in organic cations 3T^+^. Moreover, the phonon-assisted ASPL and TP-ASPL are also prominent in (2T)_2_PbI_4_ (figs. S8 and S9), implying that they are applicable to a group of 2DPKs of (2T)_2_(MA)_*m*−1_Pb*_m_*I_3*m*+1_ with different *m* values. On the basis of these results, the most possible mechanism is proposed in which the UC is enabled by strong exciton-vibrational coupling via near-band-edge states below exciton resonances in the group of these similar 2DPKs. Specifically in these materials, the near-band-edge states are available with distorted inorganic octahedral lattices (*44*–*46*), and the exciton-vibrational coupling is enhanced through the strongly π-conjugated organic cations (*42*, *47*, *48*).

### Dynamics investigation of ultrafast phonon-assisted UC

**Fig. 3.**
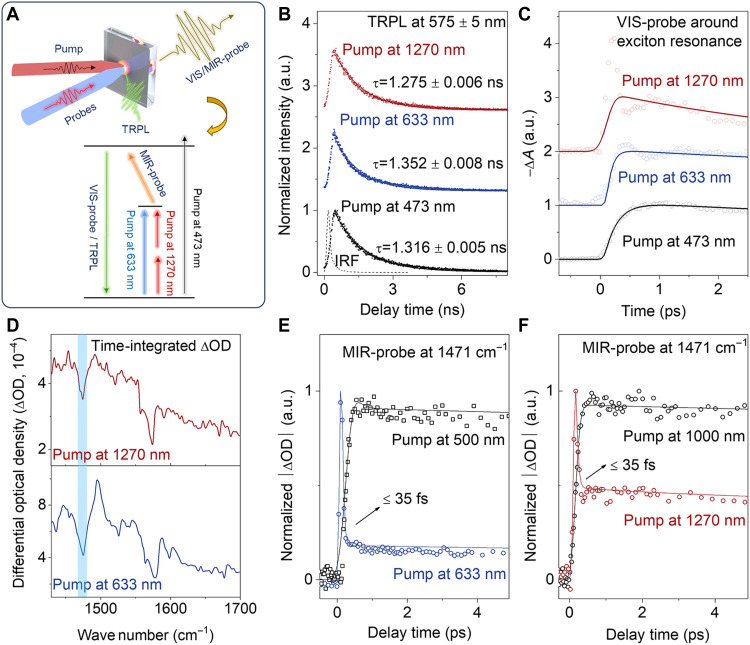
Dynamics of ASPL and TP-ASPL. (**A**) Dynamics experimental configuration with schematics of the measurement setup and energy diagrams. For various excitation conditions, TRPL spectra, TA spectra with the VIS-probe, and TA spectra with the MIR-probe are combined to elucidate the dynamics. (**B**) TRPL spectra with fitted curves under various pumps at 473, 633, and 1270 nm. The dashed curve for the pump at 473 nm represents the instrumental response function (IRF). (**C**) TA spectra with the VIS-probe around the exciton resonance at 575 nm. Note that there is an instantaneous overshoot at time zero for both excitations because of the convoluted optical Stark effect, which is omitted for the rise time fitting (*6*). (**D**) Time-integrated ΔOD spectra of TA with the MIR-probe. Among several resonances, the blue-shaded area at 1471 cm^−1^ highlights a typical one for UC dynamics. (**E** and **F**) TA spectra with the MIR-probe at 1471 cm^−1^ for above-gap and ASPL excitations at 500 and 633 nm (E) and for TP and TP-ASPL excitations at 1000 and 1270 nm (F). Both indicate the ultrafast dynamics of ≤35 fs for phonon-assisted UC. Note that all these spectra for ASPL (TP-ASPL) were taken with low excitation powers at the linear (quadratic) regime.

To demonstrate the underlying dynamic mechanisms of phonon-assisted UC, microscopic time-resolved PL (TRPL) and transient absorption (TA) measurements were combined with various excitation conditions. Figure 3A illustrates the dynamics experimental configuration (Materials and Methods), where the excitation conditions are categorized into four scenarios: the above-gap excitation pumped at 473 nm or at 500 nm for regular PL, the below-gap excitation pumped at 633 nm for ASPL, the TP excitation pumped at 1000 nm for TPA-PL, and the TP excitation pumped at 1270 nm for TP-ASPL. To explicitly elucidate all the dynamic processes, the time-resolved detections are based on three distinct techniques. Specifically, the TRPL can resolve the luminescence decay dynamics, the TA with a visible probe (VIS-probe) can reveal all the exciton dynamics upon ultrafast excitations, and the TA with a midinfrared probe (MIR-probe) can precisely distinguish the phonon dynamics from the exciton dynamics upon ultrafast excitations.

The dynamics can be then comprehensively analyzed from the decay processes and ultrafast excitations. For the decay processes, the TRPL spectra show a similar luminescence lifetime of ~1.3 ns with single-exponential fitting for all the excitation scenarios (Fig. 3B). This indicates that the decay processes are independent from the excitations. For the excitations, the TA spectra with the VIS-probe indicate nontrivial differences among all the scenarios, especially on the rise processes (Fig. 3C). Upon fitting the dynamics data, the rise time ( $\tau_{\text{rise}}$ ) of exciton band bleaching is ~(220 ± 10) fs for the pump at 473 nm. For pumps at 633 and 1270 nm, however, the fitted $\tau_{\text{rise}}$ values are estimated to be (90 ± 20) and (110 ± 10) fs, respectively, where the overshoots at the initial rising ranges are considered as convoluted optical Stark effects and not considered for the dynamics fitting (fig. S10) (*6*). This shows that the ASPL and TP-ASPL excitations are faster with the ultrafast dynamics of single-step UC, which approaches the TA resolution limit of the VIS-probe (*6*, *30*, *49*, *50*).

To visualize the single-step process of phonon-assisted UC, the ultrafast phonon dynamics with a 35-fs resolution are directly traced by TA with the MIR-probe. The probed MIR response originates from two components (Fig. 3D): One arises from organic cation vibrational resonances, which are consistent with FTIR (Fig. 1C), and the other stems from the absorption signals of photoexcited carriers within the materials (see Materials and Methods). Therefore, the transient MIR optical density (ΔOD) spectra probe the depopulation process of vibrational states in the electronic ground state before photoexcitation. Here, the resonance at 1471 cm^−1^ is taken as a typical mode for the ultrafast probe. For the regular PL and TPA excitations (pumps at 500 and 1000 nm) without UC, the MIR-probe shows a long lifetime of (212 ± 16) ps (Fig. 3, E and F). This time constant is comparable with the vibrational relaxation time in similar perovskite structures, which is attributed to the electron-phonon coupling upon above-gap excitations (*51*–*53*). Notably, for the ASPL and TP-ASPL excitations (pumps at 633 and 1270 nm), there are markedly faster processes right after the pump pulses, which are followed by slow processes of ~212 ps (Fig. 3, E and F). These faster processes reach the instrumental resolution limit of 35 fs, demonstrating that the phonon-assisted UC happens in a timescale of ≤35 fs. Notably, the emerging dynamics of ≤35 fs are markedly accelerated by about four orders of magnitude from the above-gap excitation scheme and approach a single cycle of the vibration (estimated to be ~23 fs). The sub–35-fs vibrational dynamics, together with the available near-band-edge states, are regarded as signature evidence to demonstrate the exciton-vibrational coupling in the single-step phonon-assisted UC (*6*, *49*, *54*). These ultrafast dynamics, comparable with the nonlinear optical processes with virtual states (*55*, *56*), could be aligned with TPA and lastly lead to the synergy of phonon-assisted UC and TPA.

## DISCUSSION

### Uniqueness and advantages of giant single-step UC

**Fig. 4.**
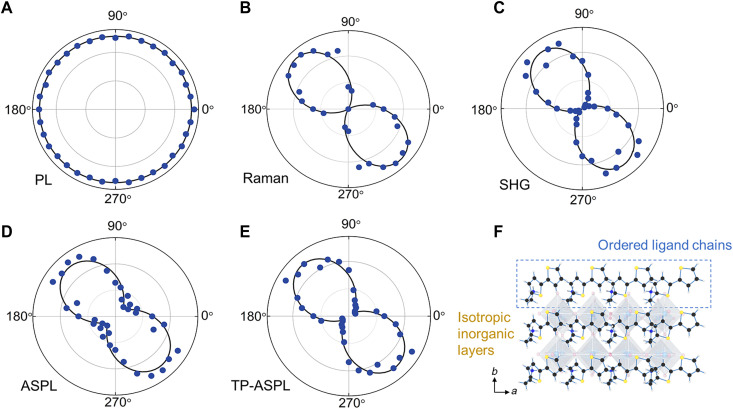
Polarization dependences of PL, Raman anisotropy, SHG anisotropy, ASPL, and TP-ASPL. (**A**) Normalized PL intensity versus polarization angle (α) in the polar coordinates when excited at 473 nm. The angle (α) refers to the angle of the collection polarization, and the excitation polarization angle is fixed. The intensity looks uniform in the polar plot without polarization dependence. (**B**) Normalized Raman intensity of 1561 cm^−1^ versus polarization angle (α) when excited at 633 nm. The Raman intensity is polarized along some specific angle, which is correlated with the ordered orientation of 2T^+^ as illustrated in (F). (**C**) Normalized SHG excited at 1270 nm. (**D** and **E**) Normalized ASPL excited at 633 nm (D) and TP-ASPL excited at 1270 nm (E). Both ASPL and TP-ASPL show the same polarization dependence with Raman anisotropy. (**F**) The simplified top view of the ordered cations in (2T)_2_(MA)Pb_2_I_7_ is sketched according to the detailed crystalline structural analysis in table S2.

To demonstrate the uniqueness of phonon-assisted UC between organic vibrations and excitons in inorganic lattices, polarization-dependent measurements were performed for PL, Raman anisotropy, SHG anisotropy, ASPL, and TP-ASPL. Here, the excitation polarization is fixed, while the collection angle is varied (see Materials and Methods). PL excited at 473 nm (Fig. 4A) shows an isotropic response without polarization dependence, while the Raman spectrum at 1561 cm^−1^ (Fig. 4B) shows a strong anisotropic response. Raman anisotropy does not depend on vibration orientations and excitation polarizations (figs. S11 and S12) but follows SHG anisotropy, which depends on the ordered orientation of the organic 2T^+^ cations (Fig. 4, C and F, fig. S4, and table S2) (*42*). This dependence indicates that the origin of Raman anisotropy is only correlated to the ordered orientation of 2T^+^ cations (Fig. 4, C and F). Different from PL, however, both ASPL excited at 633 nm and TP-ASPL excited at 1270 nm (Fig. 4, D and E) show the same anisotropic polarization dependence as Raman and SHG anisotropy. This observation manifests the same polarization origin, i.e., the orientation of organic cations (Fig. 4F and table S2). On the one hand, the octahedral inorganic structures of (2T)_2_(MA)Pb_2_I_7_ give rise to the isotropic PL of above-gap excitation. On the other hand, the organic 2T^+^ cations are highly ordered for Raman anisotropy, thus causing the anisotropic responses of ASPL and TP-ASPL. Moreover, the anisotropy of the Raman spectrum at 1073 cm^−1^, ASPL at 612 nm, and TP-ASPL at 1228 nm shows similar consistency (fig. S11). Therefore, the transition from isotropic PL to anisotropic ASPL (TP-ASPL) becomes one signature of this unique phonon-assisted UC.

Further features can be extracted by the quantitative analysis of UC quantum yield (UCQY) for ASPL and the nonlinear absorption coefficient for TP-ASPL. The UCQY can be estimated on the basis of the absorption (fig. S13) and comparative analysis (see note S2 and fig. S14). The nontrivial UCQY of 2.1% (1.6%) for ASPL at the excitation of 612 nm (633 nm) is listed in table S3, which is comparable with the UCQY of (3T)_2_(MA)Pb_2_I_7_ (fig. S15 and table S4) and other UC materials involved in the multiphonon-assisted UC of similar energy gains (table S5). As the UCQY addresses the capability of gaining energy per absorbed photon, which is limited by the electron-phonon coupling, the measured UCQY of (2T)_2_(MA)Pb_2_I_7_ further confirms the efficient electron-phonon coupling between organic vibrations and excitons in inorganic lattices. Besides, the TPA coefficient for TP-ASPL can be measured on the basis of pump power–dependent conversion efficiency measurements (fig. S16 and table S6). The nonlinear coefficient of 5.11 ± 0.05 cm/GW (5.88 ± 0.09 cm/GW) at the excitation of 1270 nm (1228 nm) is probably correlated with the TP-ASPL resonances at these near-band-edge states and enhanced exciton-phonon coupling. These values now become comparable with that of 5.64 ± 0.06 cm/GW for the above-gap TPA (excited at 1100 nm) without resonance enhancements (*57*). The quantitative analysis of the nontrivial UCQY and strong optical nonlinearity suggests that the stringent criterion has been met in this hybrid organic-inorganic 2DPK, enabling this unique synergy of phonon-assisted UC and TPA.

The advantageous exploration of ASPL and TP-ASPL with an extended spectral response, increased thermal managements, and desired nonlinear effects could directly apply to diverse photonic applications. Besides the obvious advantage of a broader spectral response, the thermal managements and nonlinear effects could be beneficial to the damage thresholds of optical materials, one of the most critical parameters for various applications. The sample damage thresholds have been tested for all the cases of ASPL, TPA, and TP-ASPL as initial demonstrations (fig. S17 and tables S7 and S8). TP-ASPL shows the highest damage threshold (table S9), which is about two orders of magnitude higher than ASPL and even several times higher than TPA. This highest damage threshold results from the synergistic nonlinear effects and optical cooling effects (*3*, *13*). Moreover, TP-ASPL also leads to the largest penetration depth of ~0.5 mm (see note S3 and figs. S13 and S17). For the applications of optical microscopy, the nonlinear effects are desired for a higher imaging resolution. The resolution can be represented by point spread function, which has also been tested for different cases (fig. S18). With the nonlinear effects, the point spread function indicates a higher spatial resolution than ASPL below the diffraction limits. These advantages bring out many possibilities for addressing challenges in optical refrigeration, upconverting photovoltaics with a broader spectral response and longer sustainability, nonlinear optical microscopy with improved thermal managements, etc., which may not be possible in other UC materials.

In this work, we present a distinctive ASPL paradigm of phonon-assisted UC in the nonlinear regime of TPA in (2T)_2_(MA)Pb_2_I_7_, which is applicable to the group of (2T)_2_(MA)_*m*−1_Pb*_m_*I_3*m*+1_ compounds with varying *m* values. Notably, to the best of our knowledge, TP-ASPL has not been found in any other materials, highlighting the unique electron-phonon coupling between organic vibrations and exciton resonances in inorganic lattices of these 2DPKs. This electron-phonon coupling, responsible for the giant single-step UC of ~200 meV within sub–35-fs phonon dynamics, is demonstrated through a combination of spectroscopic measurements, material characterizations, and theoretical calculations. In particular, the organic vibrations, although decoupled from the inorganic lattice, are found to play an important role in facilitating the UC via efficient exciton-vibrational coupling. While a detailed mechanism of this coupling merits further investigations of more comprehensive dynamics measurements, the established single-step UC gives rise to TP-ASPL with synergistic features absent in conventional ASPL. With the established mechanism of phonon-assisted UC, TP-ASPL manifests synergistic features that are absent in conventional ASPL. Intriguingly, the synergy of phonon-assisted UC and TPA offers substantial advantages, such as a broader spectral response, improved thermal managements, and desired nonlinear effects, paving the way for advanced photonic applications beyond the limits of traditional ASPL. As 2DPKs materials continue to be extensively explored for photonics and optoelectronics, the realization of TP-ASPL–based applications may soon be within reach.

## MATERIALS AND METHODS

### Sample preparation

2T·hydroiodic acid (HI; 17.8 μmol; 98%, Shenzhen Feiming Science and Technology Co., Ltd.), 126 μmol of methylammonium iodide (MAI; 99.99%, Greatcell Solar Ltd.), 4.3 μmol of lead iodide (PbI_2_; 99.999%, Xi’an Yuri Solar Co., Ltd.), 0.1 ml of HI (57 wt %, TCI), 0.05 ml of hypophosphorous acid (H_3_PO_2_; J&K Scientific), and 2 ml of isopropyl alcohol (Sinopharm) were added in a glass vial. The solid precursors were completely dissolved in the mixed solvent by heating the capped glass vial in an oil bath to 110°C. After that, it was quickly transferred to a muffle furnace and slowly cooled down to room temperature at a cooling rate of 2°C/hour. Last, macroscopically sized red crystals (2T)_2_(MA)Pb_2_I_7_ were obtained and collected. For measurement purposes, the bulk crystals were mechanically exfoliated onto SiO_2_/Si substrates.

For the (2T)_2_PbI_4_ single crystal, 2T·HI (0.03 mmol, 98%, Shenzhen Feiming Science and Technology Co., Ltd.) and PbI_2_ (0.01 mmol, PbI_2_, 99.999%, Xi’an Baolai Photoelectric Technology Co., Ltd.) were dissolved in HI (0.1 ml), H_3_PO_2_ (0.05 ml), and isopropyl alcohol (2 ml). For (3T)_2_MAPb_2_I_7_, 3T·HI (0.024 mmol, 98%, Shenzhen Feiming Science and Technology Co., Ltd.), methylammonium iodide (0.5 mmol, 99.99%, Greatcell Solar Materials), and PbI_2_ (0.16 mmol) were dissolved in HI (1 ml), H_3_PO_2_ (0.5 ml), and ethanol (EtOH; 7.5 ml). After heating to 100°C, the solution became clear. It was then cooled slowly to room temperature for 48 hours to obtain a high-quality single crystal.

### DFT calculation of phonon modes for 2T^+^ cations

The hybrid exchange-correlation functional B3LYP was used to optimize all structures, and the basis set 6-311+G(d,p) was adopted for the atoms to calculate the energy for a number of conformations of 2T^+^ cations of the 2DPK of (2T)_2_(MA)Pb_2_I_7_. The first-principle DFT calculations were performed using the Gaussian 16 program (*58*). Figure S2 displays the calculated structures of the 2T^+^ cations only, which correspond to the minimum and maximum energy values found. Vibrational frequency calculations show that all the optimized structures are minima on the potential energy surface of ground states. The Raman intensities were calculated by using a differential Raman scattering cross section. The calculated harmonic frequencies were scaled by scaling factors of 0.981 for below 2000 cm^−1^ and 0.967 for above 2000 cm^−1^. In this case, the Raman scattering intensity was calculated using

$$\begin{array}{c} I_{\text{Raman}}=\frac{{\left(2\pi \right)}^{4}}{45}\frac{h}{8\pi^{2}c\omega_{i}}\frac{{\left(\omega_{0}-\omega_{i}\right)}^{4}}{1-\text{exp}\left(-\frac{\mathrm{hc}\omega_{i}}{k_{B}T}\right)}\left[45{\left(\frac{d\alpha }{dQ_{i}}\right)}^{2}+7{\left(\frac{\mathrm{dy}}{dQ_{i}}\right)}^{2}\right]\\ \;=\frac{{\left(2\pi \right)}^{4}}{45}\frac{h}{8\pi^{2}c\omega_{i}}\frac{{\left(\omega_{0}-\omega_{i}\right)}^{4}}{1-\text{exp}\left(-\frac{\mathrm{hc}\omega_{i}}{k_{B}T}\right)}S_{i} \end{array}$$ (1)

where *h*, *c*, *k*_B_, and *T* are the Planck constant, light speed, Boltzmann constant, and kelvin temperature, respectively. $S_{i}$ is the Raman scattering factor (in Å^4^/amu) that can be calculated using Gaussian 16 at the equilibrium geometry, and it is only an expression of derivatives in the static isotropic and anisotropic polarizabilities with respect to the given normal coordinate. Here, ω_0_ and ω*_i_* denote the frequency (in cm^−1^) of the incident light and vibrational frequency of the *i*th mode, respectively. The simulated Raman spectra were presented in terms of a Lorentzian expansion with a linewidth of 10 cm^−1^. The differential Raman scattering cross-sectional values of different vibrational modes were calculated from the Raman scattering factor under the double-harmonic approximation, with a specific excitation wavelength of 633 nm at room temperature.

### Optical measurements

Most of the optical measurements were carried out at room temperature, unless noted otherwise. PL spectroscopy was carried out by a continuous-wave laser at 473 nm and a femtosecond laser (PHAROS, Light Conversion) for comparison. For the excitations in ASPL and TP-ASPL, the wavelengths of the femtosecond laser (PHAROS, Light Conversion) were scanned from 595 to 1308 nm. The Raman spectrum was excited by a continuous-wave laser (cnilaser, MDL-E-633) at 633 nm and collected by either a commercial Horiba Jobin Yvon spectrometer (XploRA plus) or homebuilt setup integrated with an Andor spectrometer. In the homebuilt setup, the laser was focused and the signals were collected with a microscope objective (magnification of 100× and numerical aperture of 0.9 for Raman anisotropy, PL, ASPL, and TP-ASPL). The polarization dependence for the homebuilt setup was realized by a half-wave plate and linear polarizer in both the optical paths of excitation and detection. The angles of the polarization dependence were set by rotating the polarization from an initial angle (α) through α + 180°. The data at the polarization angles from α + 180° through α + 360° were mirrored from those at angles from α through α + 180° (by a LBTEK rotation mount). FTIR spectroscopy was carried out by a commercial spectrometer of Thermo Fisher Scientific (Nicolet iN10).

Time-resolved spectroscopy includes TRPL, TA with the VIS-probe, and TA with the MIR-probe. In TRPL, samples were excited by the femtosecond laser (PHAROS, Light Conversion), and the signals were detected by single-photon avalanche diodes of the PDM Series connected to a time-correlated single-photon counting system from PicoQuant. The lifetimes were fitted without considering the convolution time range around the intensity peak and then approximated by a single exponential as $I=I_{0}e^{-\left(t-t_{0}\right)/\tau }$ , where *I*_0_ is the initial intensity at *t* = $t_{0}$ , and τ is the lifetime.

For the TA, the VIS-probe experiment uses laser pulses (Spitfire Ace, Spectra-Physics, 800 nm, 5 kHz, 70 fs) and drives a commercial tunable optical parametric amplifier (TOPAS) to generate tunable femtosecond pulses in the ultraviolet–to–near-infrared band as pump light for the sample. The MIR-probe experiment uses laser pulses from a titanium-sapphire femtosecond laser (35 fs, 800 nm, 1 kHz) to pump an optical parametric amplifier (TOPAS Prime, Spectra Physics). Changes in the visible detection light during the experiment are collected using two fiber optical spectrometers (AvaSpec-ULS2048CL-EVO and AvaSpec-NIR256-1.7-HSC-EVO, Avantes), while changes in the MIR detection light are collected using a 64-pixel cadmium mercury telluride MIR spectrometer (FPAS-0144, Infrared Systems Development Corporation). The observed MIR response originates from two components: One arises from organic cation vibrational resonances, and the other stems from the absorption signals of photoexcited carriers within the materials. The latter is the source of the observed broad photoinduced absorption signal, which exhibits an absorption background across nearly the entire MIR spectral window. The negative peaks observed on top of this absorption background indicate a reduction in the absorption of vibrational resonances corresponding to a depopulation of vibrational states. Specifically, transient MIR spectroscopy probes the depopulation process of vibrational states in the electronic ground state before photoexcitation. The probed signals are defined as the MIR absorption changes between a given delay time and the preexcitation time (*t* = −1 ps) (ΔOD = OD_*t*=−1ps_ − OD*_t_*), representing a time-dependent depopulation of vibrational states. This depopulation results from the coupling between the local vibrations of the organic cations and the excitons within the inorganic lattice, which occurs within 35 fs for the UC process.

## Supplementary Materials

### This PDF file includes:

Notes S1 to S3 Figs. S1 to S18 Tables S1 to S9 References
