# Supplementary material for: Giant single-step upconversion via sub–35-fs phonon dynamics in the nonlinear optical regime
Source: Sci Adv. 2025 Oct 15;11(42):eadx1686. doi: 10.1126/sciadv.adx1686 (PMC12525952; doi:10.1126/sciadv.adx1686)
Supplement: Supplementary file 1 — Notes S1 to S3 Figs. S1 to S18 Tables S1 to S9 References [file sciadv.adx1686_sm.pdf]

Science Advances
AAAS

Supplementary Materials for

Giant single-step upconversion via sub–35-fs phonon dynamics in the nonlinear optical regime

Jingjing Yao *et al.*

Corresponding author: Hailong Chen, hlchen@iphy.ac.cn; Enzheng Shi, shienzheng@westlake.edu.cn; Xiaoze Liu, xiaozeliu@whu.edu.cn

Sci. Adv. **11**, eadx1686 (2025)
DOI: 10.1126/sciadv.adx1686

This PDF file includes:

Notes S1 to S3
Figs. S1 to S18
Tables S1 to S9
References

Notes

1. Temperature dependence of ASPL intensity governed by phonon occupation number

To clarify the physical origin of the Arrhenius-like temperature dependence observed in Figs. 2D and 2H of the main text, we provide a detailed discussion here based on phonon occupation statistics.

For phonon-assisted UC involving the absorption of a single phonon with energy $\hbar\omega$, the ASPL intensity is expected to scale with the phonon occupation number $n\ (\omega, T)$, which follows Bose-Einstein statistics:

$$n(\omega, T) = \frac{1}{exp(\hbar\omega/k_B T)-1} \quad \text{(Eq. S1)}$$

In the limit where $\hbar\omega \gg k_B T$, which applies to our system ($\hbar\omega \approx$ 130–197 meV, $k_B T \approx$ 25 meV at room temperature), the exponential term dominates, and the occupation number can be approximated as:

$$n(\omega, T) \approx \exp\left(-\frac{\hbar\omega}{k_B T}\right) \quad \text{(Eq. S2)}$$

This approximation yields an Arrhenius-like temperature dependence for ASPL intensity:

$$I_{\mathrm{ASPL}} \propto n(\omega, \mathrm{T}) = \exp\left(-\frac{\hbar\omega}{k_B T}\right) \quad \text{(Eq. S3)}$$

Accordingly, ln (I_{ASPL}/I_{PL}) vs. 1/T yields a linear dependence with a slope corresponding to the phonon energy, which is consistent with the data and fits shown in the main text. The extracted $\hbar\omega$ from the fits (158 ± 36 meV and 196 ± 31 meV) align well with the phonon energies identified from vibrational spectroscopies (Raman/FTIR), reinforcing the assignment of a single-step, single-phonon absorption process.

2. Determination of photoluminescence quantum yields (PLQY) of $(2T)_2MAPb_2I_7$

PL quantum yields (PLQY) of $(2T)_2MAPb_2I_7$ can be determined by comparative analysis with standard quantum dots. The cadmium-based photoluminescent quantum dots (PL: 570-580 nm, Concentration: 25 mg/mL, Solvent: Chlorobenzene, by Suzhou Starlight Nanotechnology Co., Ltd.) were selected as a reference for PLQY. The quantum dots were first spin-coated onto quartz slides, and the absolute PLQY of the quantum dots excited at 450 nm was measured to be 65.5% based on the radiance of light emitted from the integrating sphere for absorption and emission measurements(*11*).

According to the definition, the PLQY can be obtained as

$$N_{PL} = N_{abs} \times \phi_{PL} \quad \text{(Eq. S4)}$$

where N_{PL}, N_{abs} are the total photons of PL and absorption, respectively; ϕ_{PL} is the PLQY of the sample.

By taking the quantum dots as a standard reference, the PLQY of tested sample ϕ_{sample} can be obtained as:

$$\frac{N_{quantum\ dots}}{N_{sample}} = \frac{N_{quantum\ dots-abs}}{N_{sample-abs}} \times \frac{\phi_{quantum\ dots}}{\phi_{sample}} \quad \text{(Eq. S5)}$$

where $N_{quantum\ dots}$, $N_{quantum\ dots-abs}$ are the total photons of PL and absorption by the quantum dots, respectively; N_{sample}, $N_{sample-abs}$ are the total photons of PL and absorption by the tested sample, respectively

After obtaining the PLQY for the sample, the upconversion (UC) PLQY (UCQY) of ϕ_{ASPL} can be calculated by following Eq. S4.

$$\phi_{\mathrm{ASPL}} = \phi_{\mathrm{PL}} \times \frac{N_{\mathrm{ASPL}}}{N_{\mathrm{PL}}} \times \frac{N_{\mathrm{PL-abs}}}{N_{\mathrm{ASPL-abs}}} \quad \text{(Eq. S6)}$$

where $\boldsymbol{N_{ASPL}}$, $\boldsymbol{N_{ASPL-abs}}$ are the total photons of ASPL and absorption by the excitation laser for the ASPL, respectively; $\boldsymbol{N_{PL}}$, $\boldsymbol{N_{PL-abs}}$ are the total photons of PL and absorption by the excitation laser for the regular PL, respectively.

The conversion efficiency of TP-ASPL can be analyzed by TPA. In the nonlinear process with TPA, the incident laser intensity (*I*) is strongly dependent on the penetration depth of *z* in the tested sample and can be described by(*59, 60*)

$$\frac{dI}{dz} + \alpha I + \beta I^2 = 0 \quad \text{(Eq. S7)}$$

where α and β are one- and two-photon absorption coefficients, respectively. Solving equation (Eq. S7) yields

$$I(z) = \frac{\alpha I(0) e^{-\alpha z}}{\alpha + \beta I(0)(1 - e^{-\alpha z})} \quad \text{(Eq. S8)}$$

where I (0) is the incident intensity of excitation light. In the case where linear absorption is negligible, i.e., $\alpha \ll 1$. The Eq. S8 can be expressed as

$$I(z) = \frac{I(0) e^{-\alpha z}}{1 + \beta I(0) z} \quad \text{(Eq. S9)}$$

The transmittance of the medium can be expressed as(*60-63*)

$$T = \frac{I(z)}{I(0)} = \frac{e^{-\alpha z}}{1 + \beta I(0) z} = \frac{T_0}{1 + \beta I(0) z} = T_0 T_N \quad \text{(Eq. S10)}$$

where T_0 is linear transmittance, T_N is nonlinear transmittance. We set the incident light intensity as I (0) = I_0, with an effective length of $z = L$.

$$T_N = \frac{1}{1 + \beta I_0 L} \quad \text{(Eq. S11)}$$

In the case of two-photon saturation absorption, the two-photon absorption of $(2T)_2MAPb_2I_7$ nanocrystals is applicable to the uniform broadening model.

$$\beta_A = \frac{\beta_0}{1 + \frac{I^2}{I_S^2}} \quad \text{(Eq. S12)}$$

where β_0 is nonsaturation TPA coefficient (a constant), TPA saturation intensity I_s.

3. Penetration depths of $(2T)_2MAPb_2I_7$ for PL, ASPL and TP-ASPL

The penetration depth can be calculated from the absorption coefficient as(*64*)

$$\delta_p = \frac{-\ln 0.1}{2\alpha} = \frac{2.3}{2\alpha} \quad \text{(Eq. S13)}$$

where δ_p is the penetration depth and α is the absorption coefficient. The absorption coefficient can be determined using the relation

$$\alpha = \frac{4\pi k}{\lambda} \quad \text{(Eq. S14)}$$

where k is the extinction coefficient and λ is the wavelength of the laser. Therefore, the relationship between penetration depth and extinction efficient can be obtained as

$$d_p = \frac{2.3\lambda}{8\pi k} \quad \text{(Eq. S15)}$$

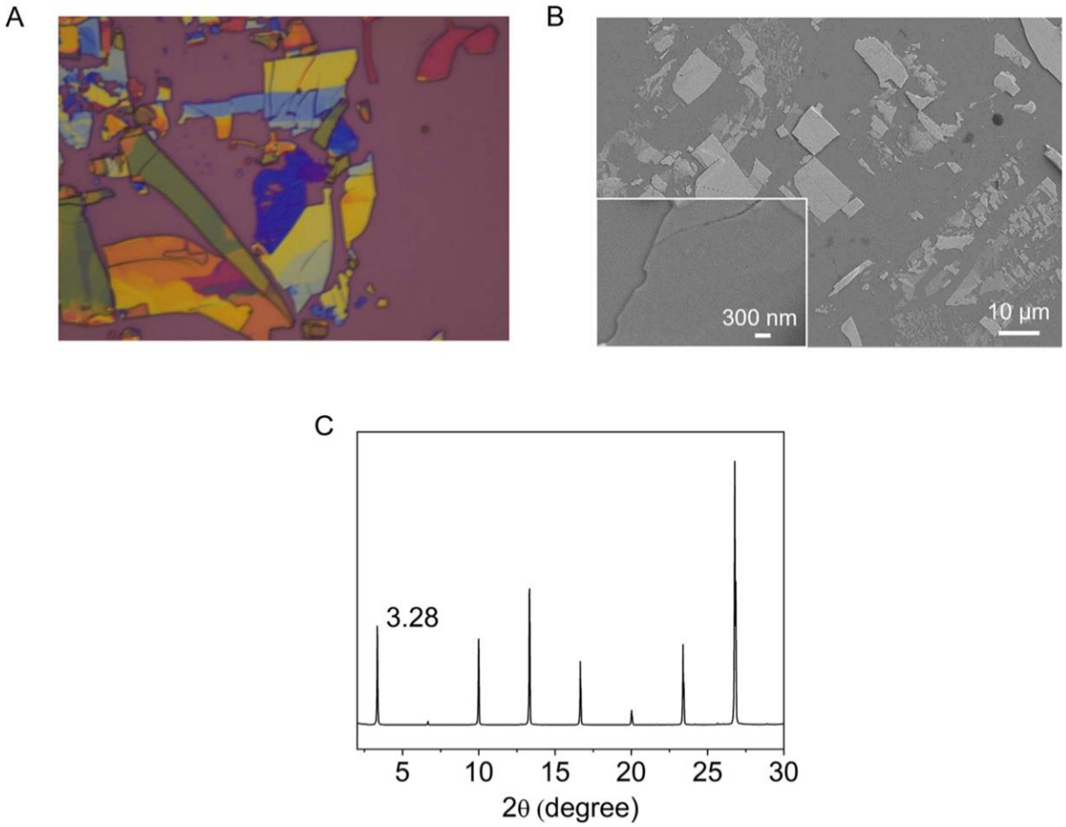

Fig. S1. Characterizations of two-dimensional perovskite (2DPK) of $(2T)_2(MA)Pb_2I_7$. (**A**) Optical microscopic image of exfoliated $(2T)_2(MA)Pb_2I_7$ on a SiO_2/Si substrate. (**B**) Scanning electron microscopic (SEM) image of the exfoliated samples. Inset is a zoomed-in image to show smooth surface morphology of $(2T)_2(MA)Pb_2I_7$. (**C**) Out-of-plane X-ray diffraction (XRD) spectrum for the $(2T)_2(MA)Pb_2I_7$.

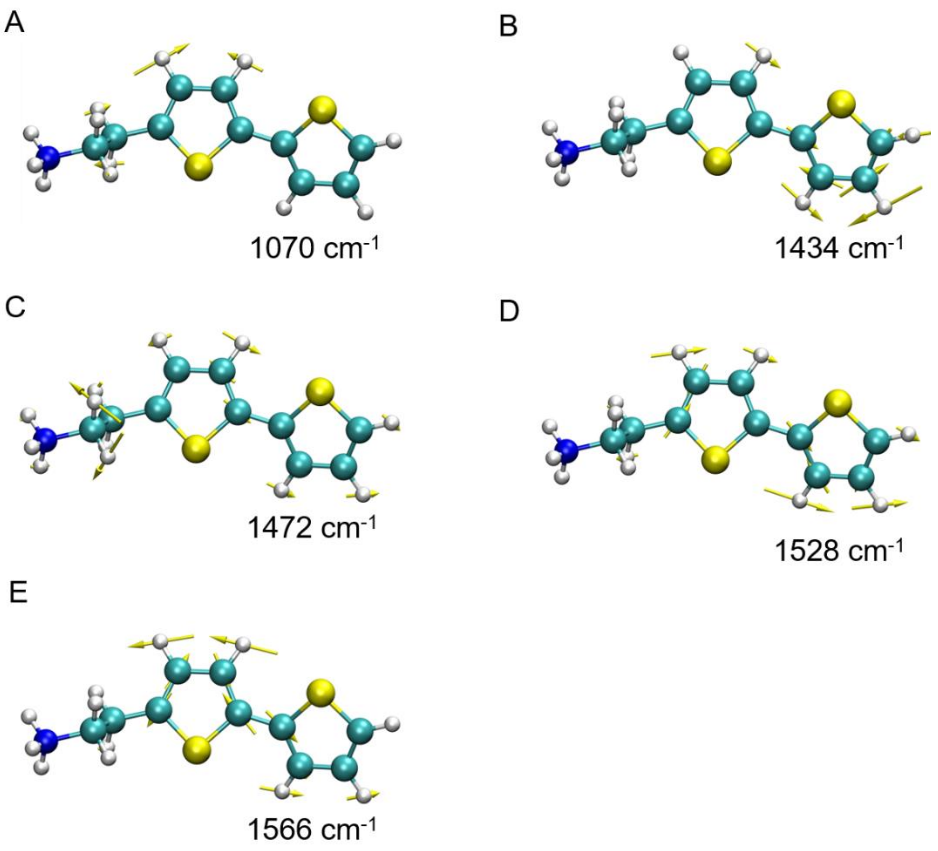

Fig. S2. Density functional theory (DFT) calculations of the vibration modes in $2T^+$. Optimized geometrical $2T^+$ cations of $(2T)_2(MA)Pb_2I_7$ (1 monomer unit) is shown as the atomic configurations in **A-E** (calculation details are elaborated below). The white, blue, green, and yellow spheres represent hydrogen, nitrogen, carbon, and sulfur atoms, respectively. (**A-E**) illustrates different vibrations based on DFT calculations for the $2T^+$ cations only, which correspond to the simulated geometrical structure and measured phonon modes in Fig. 1.

It is worth noting that within the vibration range of 1000-1700 cm^{-1}, there are five characteristic vibration frequencies (**A-E**). These frequencies correspond to, in increasing order, the stretching and bending vibrations of the C-H bonds, the C-C stretch of thiophene, C-H rocking and scissor bending mode of H-C-H, C-C antisymmetric stretch of thiophene far away from alkyl chain, C-C antisymmetric stretch of thiophene which close to alkyl chain, respectively. In comparisons with experimental measurements, these calculated vibrations are attributed to the peaks at (**A**) 1070 cm^{-1}, (**B**)1434 cm^{-1}, (**C**)1472 cm^{-1}, (**D**)1528 cm^{-1}, and (**E**) 1566 cm^{-1} as in Fig. 1.

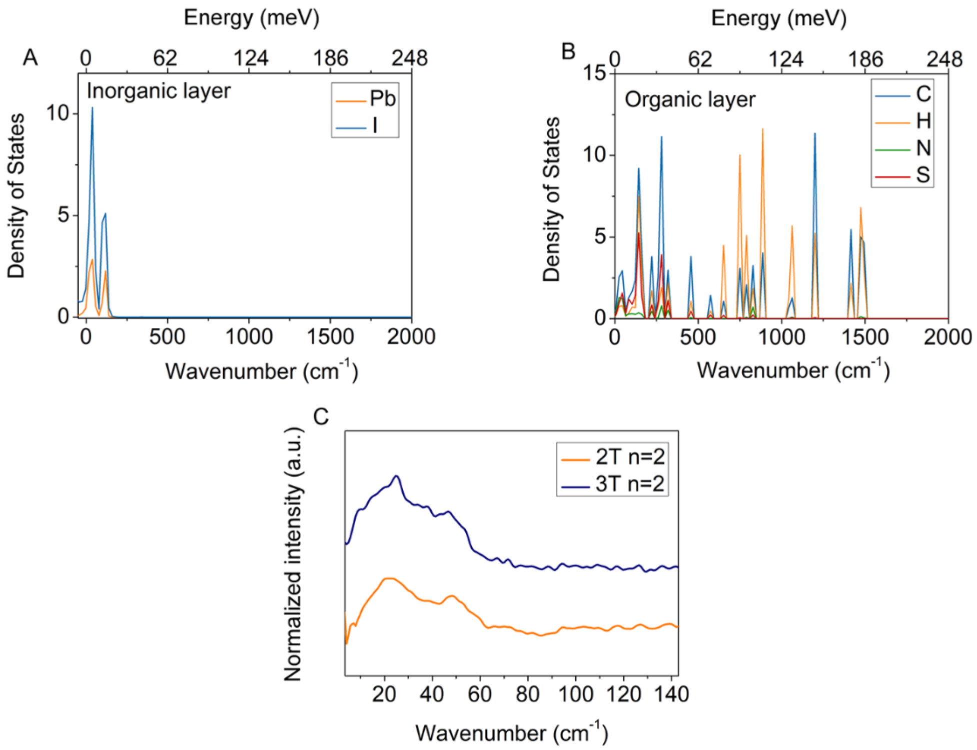

Fig. S3. Calculated phonon density of states (PDOS) for $(2T)_2(MA)Pb_2I_7$ and low-frequency Raman spectroscopy of 2DPK. (**A**) Calculated PDOS of the collective lattice phonons in the inorganic Pb-I lattices. (**B**) Calculated PDOS of the local vibrations in the organic cations. (**C**) low-frequency Raman spectra of $(2T)_2(MA)Pb_2I_7$ and $(3T)_2(MA)Pb_2I_7$ with similar lattice structures. Here $3T^+$ represents 2-([2,2':5',2''-terthiophen]-5-yl)ethan-1-aminium]).

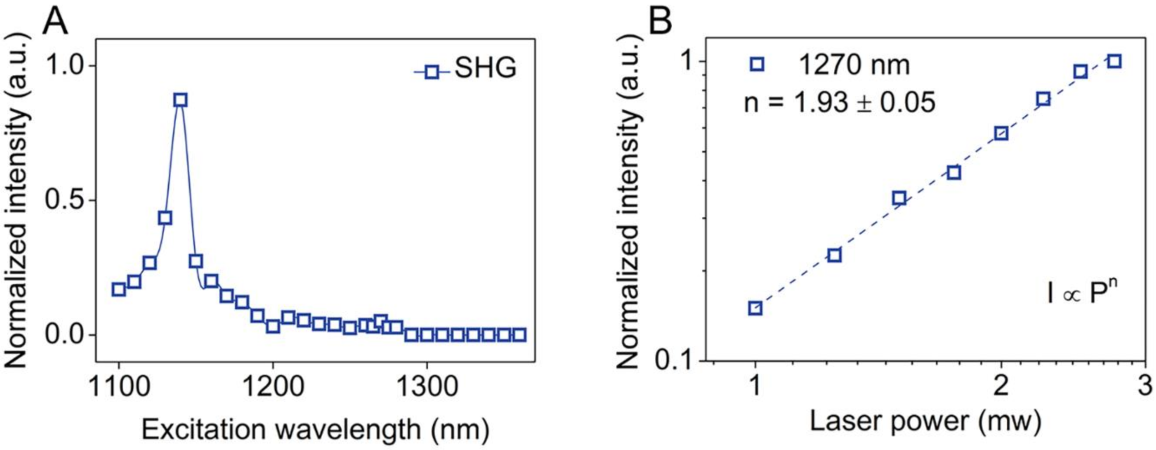

Fig. S4. Evolution of the SHG spectra as a function of the laser excitation wavelength in $(2T)_2(MA)Pb_2I_7$. (**A**) Excitation wavelength is scanned from 1100 nm to 1360 nm at room temperature. The pump power is 1 mW. (**B**) Power dependence of the SHG at the pump wavelength of 1270 nm. The power index is close to 2.

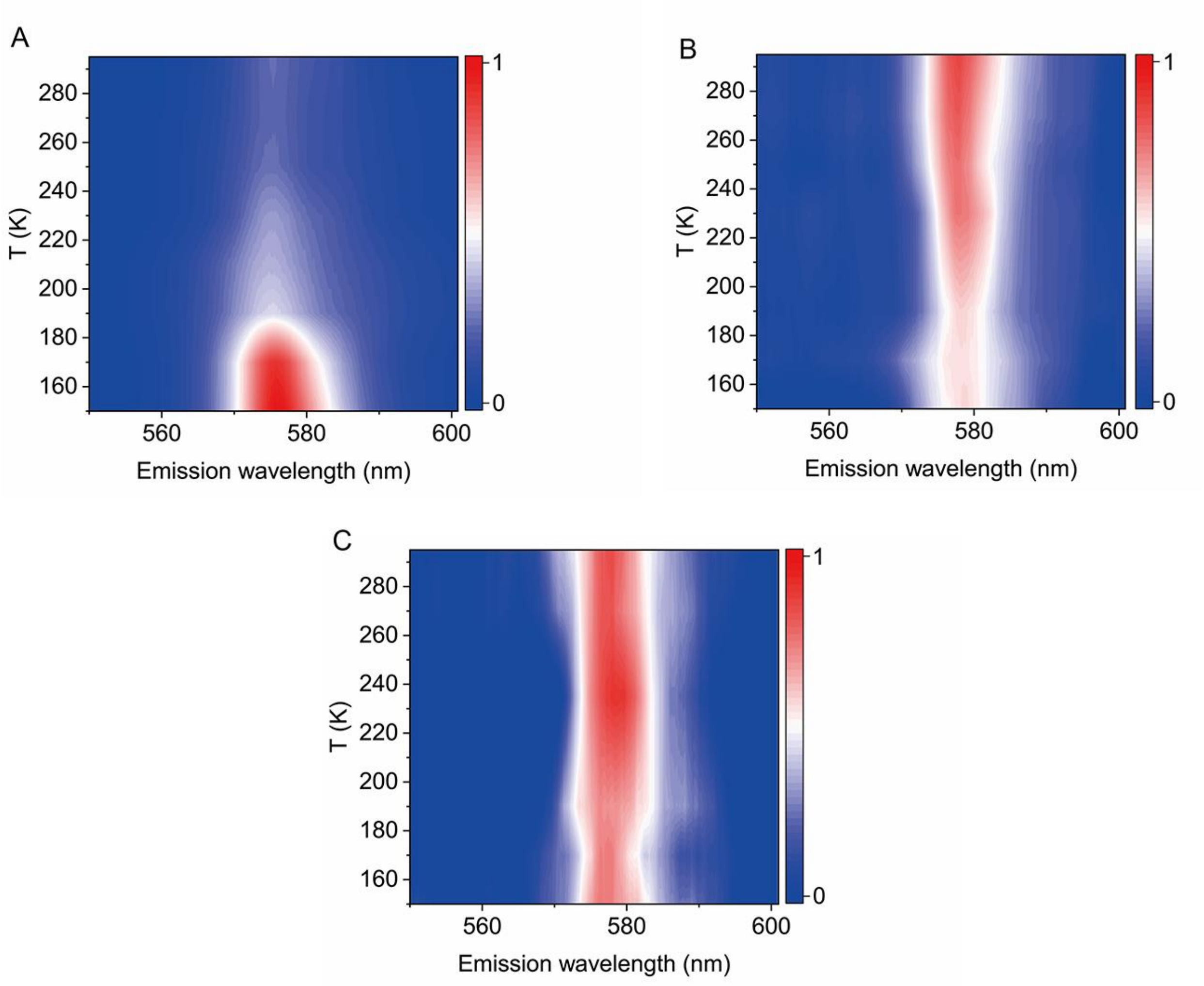

Fig. S5. PL spectra of $(2T)_2(MA)Pb_2I_7$ at different temperatures with various excitations. The spectra for (**A**) Pump at 473 nm, (**B**) Pump at 612 nm, and (**C**) Pump at 633 nm.

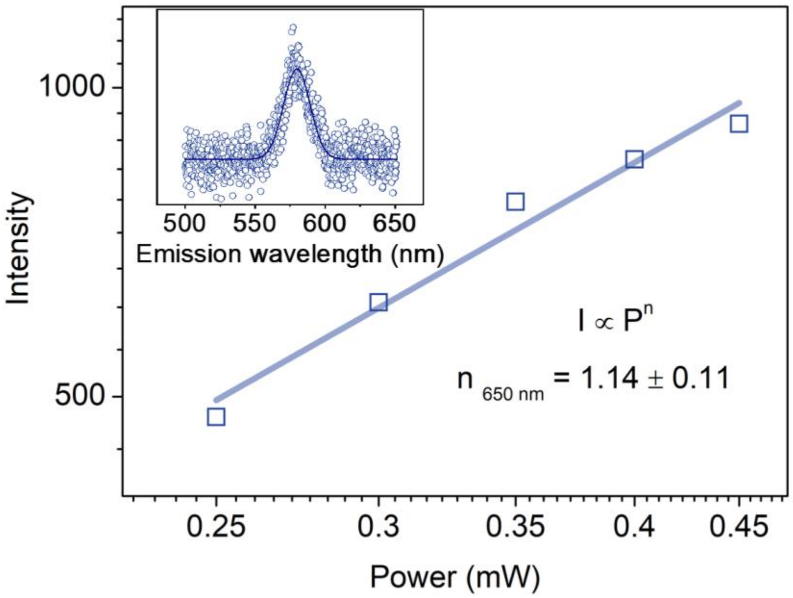

Fig. S6. Power dependence of the ASPL in $(2T)_2MAPb_2I_7$ excited at 650 nm. Based on the power-law fitting, the power index is close to 1, indicating a one-photon excitation process.

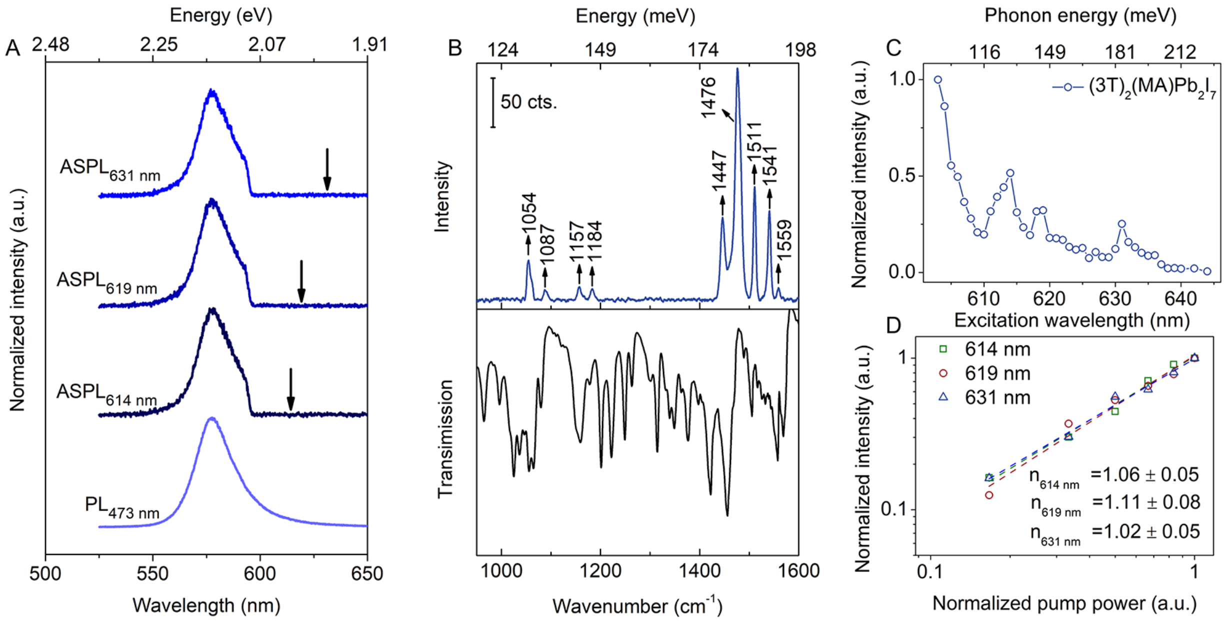

Fig. S7. ASPL characterization in $(3T)_2(MA)Pb_2I_7$. ($3T^+$: 2-([2,2':5',2''-terthiophen]-5-yl)ethan-1-aminium]) (**A**) PL and ASPL spectra of $(3T)_2(MA)Pb_2I_7$. All the spectra peak at 577 nm with similar profiles. The excitations of ASPL at 614 nm, 619 nm and 633 nm are indicated by the arrows, respectively. (**B**) Phonon characterizations of $(3T)_2(MA)Pb_2I_7$. Top panel: Raman spectrum excited at 785 nm; bottom panel: FTIR spectrum. (**C**) Excitation spectrum for ASPL where the detection is parked at the PL peak of 577 nm and the excitation is scanned from 603 nm to 644 nm with a fixed power of 6 μW. The excitation spectrum peaks at 614 nm, 619 nm and 631 nm. (**D**) Pump-power dependences of ASPL under different excitations. The power dependences of the ASPL excited at 614 nm, 619 nm and 631 nm show power indices ~ 1 for one-photon excitation. The UC gains between excitation resonances and PL peak are obtained to be (130 ± 17) meV, (146 ± 17) meV and (184 ± 16) meV.

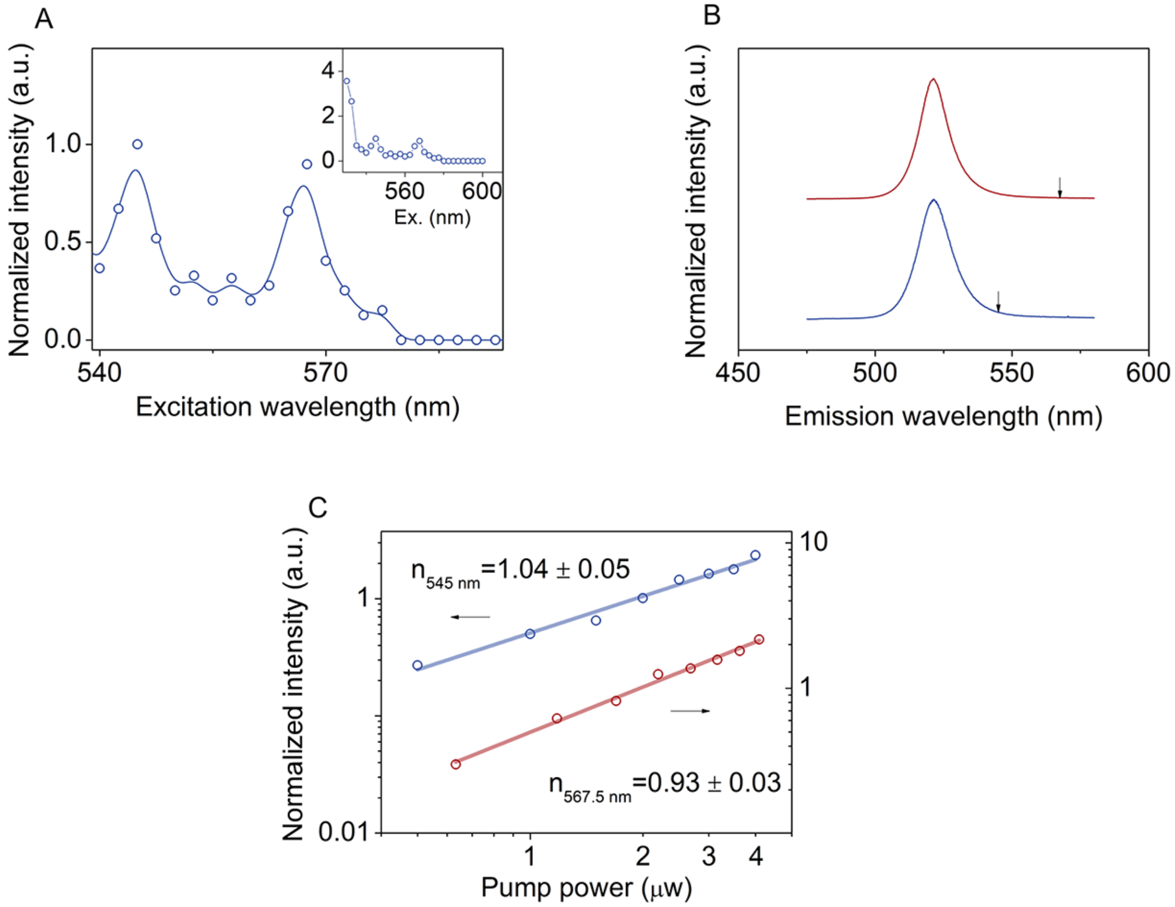

Fig. S8. ASPL excitation spectrum for $(2T)_2PbI_4$ with layer number n = 1. (**A**) The detected intensity is parked at the PL peak of 520 nm and the excitation wavelength is scanned from 530 nm to 600 nm (full range in the inset) with a fixed power of 1.3 μw. In the zoomed-in range, there appear two peaks appear around 545 nm and 567.5 nm. The energy differences between excitation and emission peak are obtained to be (110 ± 21) meV and ~ (200 ± 19) meV, which are roughly consistent with the presented case of $(2T)_2(MA)Pb_2I_7$ with n = 2 in the main text. (**B**) The ASPL spectra excited at 545 nm and 567.5 nm. (**C**) The power-dependent ASPL excited at 545 nm and 567.5 nm. The power law fitting for both ASPL shows the power indices are ~1, indicating single-photon excitation process with phonon-assisted upconversion.

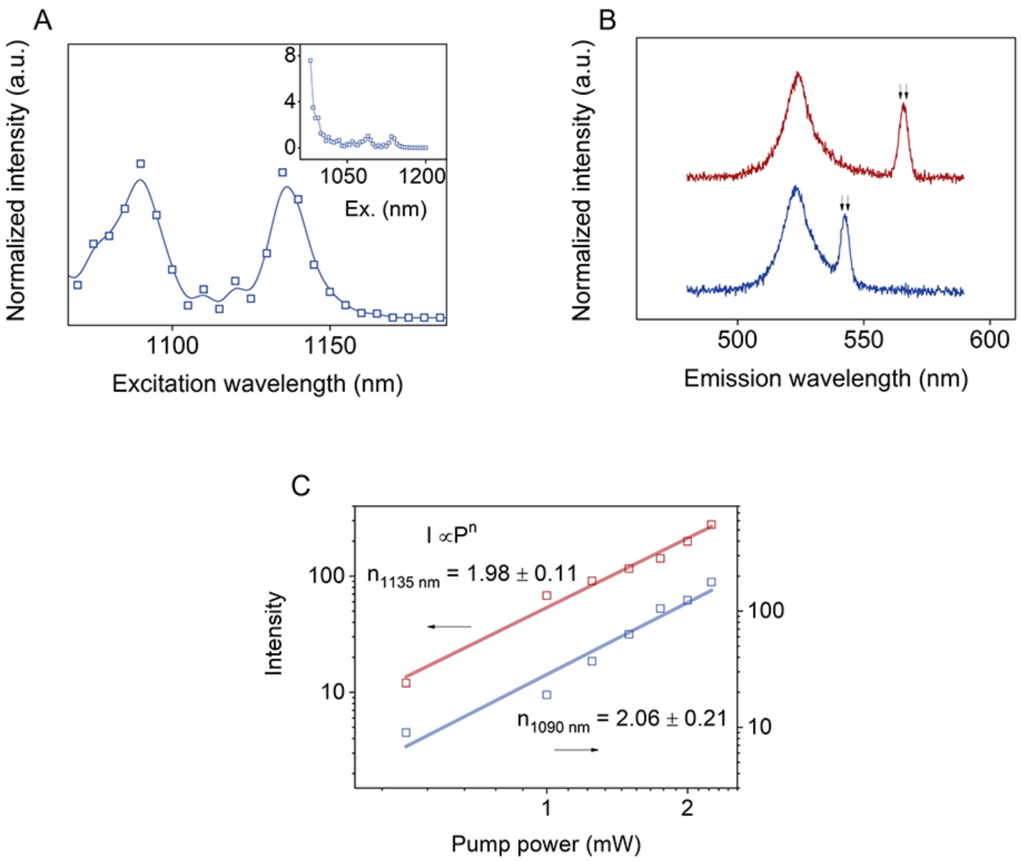

Fig. S9. TP-ASPL excitation spectrum for $(2T)_2PbI_4$ with n=1. (**A**) The detected intensity is parked at the PL peak of 520 nm and the excitation wavelength is scanned from 980 nm to 1200 nm (full range in the inset) with a fixed power of 1 mw. In the zoomed-in range, there appear two peaks appear around 1090 nm and 1135 nm. (**B**) The TP-ASPL spectra excited at 1090 nm and 1135 nm. Other than the PL peak around 525 nm, there is another peak in both cases corresponding to the second harmonic generation (SHG). (**C**) The power-dependent TP-ASPL excited at 1090 nm and 1135 nm. The power law fitting for both TP-ASPL shows the power indices are ~2, indicating two-photon nonlinear process with phonon-assisted upconversion.

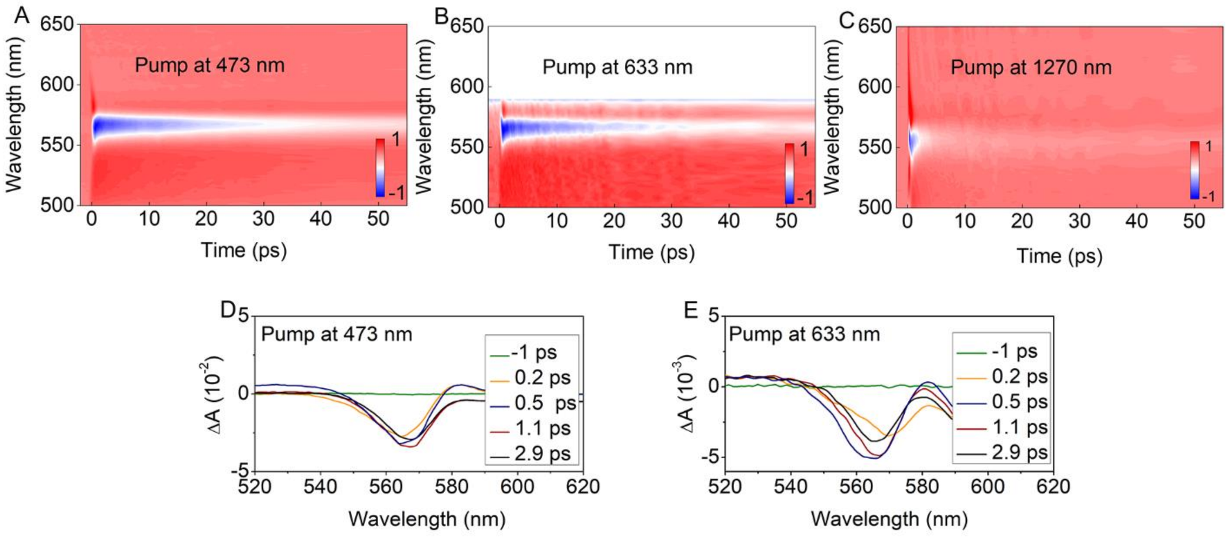

Fig. S10. Transient absorption (TA) measurements of $(2T)_2(MA)Pb_2I_7$. All these spectra are taken under low excitation powers at the linear (quadratic) range for the ASPL (TP-ASPL). (**A-C**) The TA measurements with visible-probe for excitations at 473 nm (PL excitation) (**A**), at 633 nm (ASPL excitation) (**B**) and at 1270 nm (TP-ASPL excitation) (**C**) are similar after the first few ps upon excitations. For the first few ps, there is an instantaneous overshoot at delay time zero for both ASPL and TP-ASPL excitations due to the convoluted optical Stark effect. To roughly extract the rise times for simplified comparisons, this overshoot of convoluted optical Stark effect could be omitted as discussed in Ref (*6*). Transient spectra at selected delay times for excitations (**D**) at 473 nm, (**E**) at 633 nm.

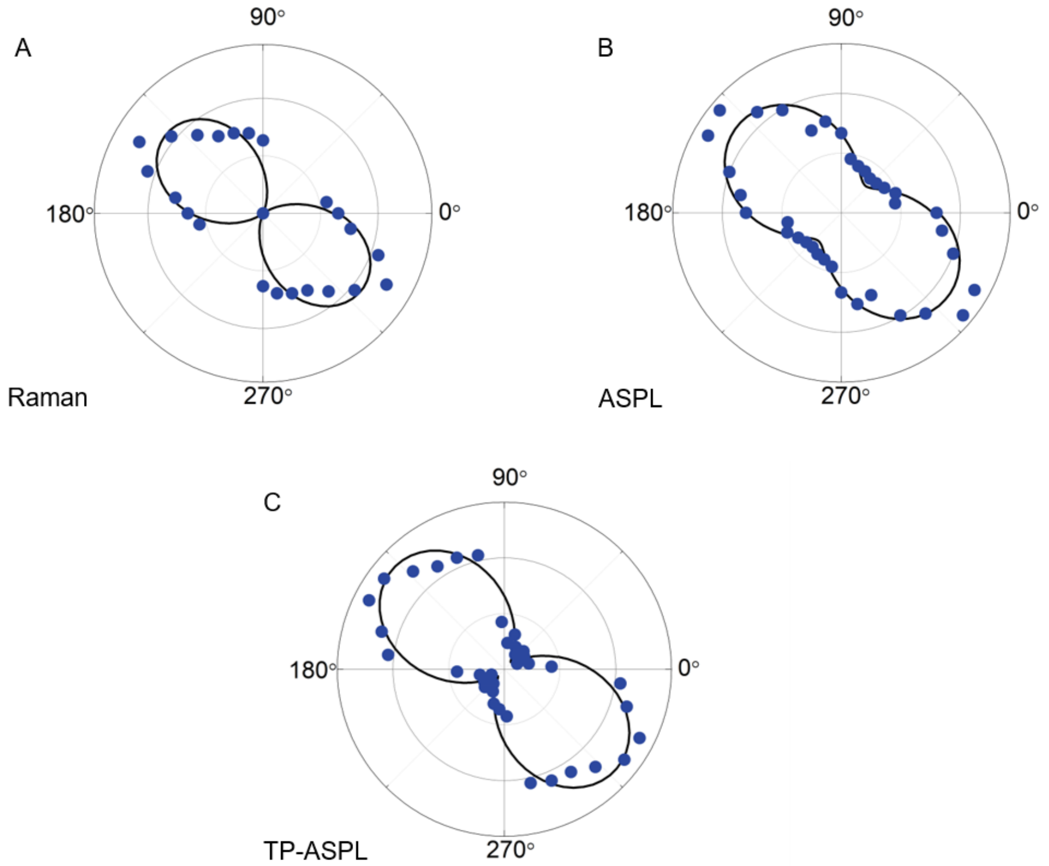

Fig. S11. Normalized Raman intensity of 1073 cm^{-1} versus polarization angle (α), and the excitation polarization is fixed at some specific angle. (**A**)The Raman intensity is polarized along ~ 135 degrees. Normalized ASPL with excitation wavelength of 612 nm (**B**) and TP-ASPL with excitation of 1228 nm (**C**). Both ASPL and TP-ASPL show the same polarization dependence with the Raman intensity, confirming the phonon-assisted upconversion for both scenarios.

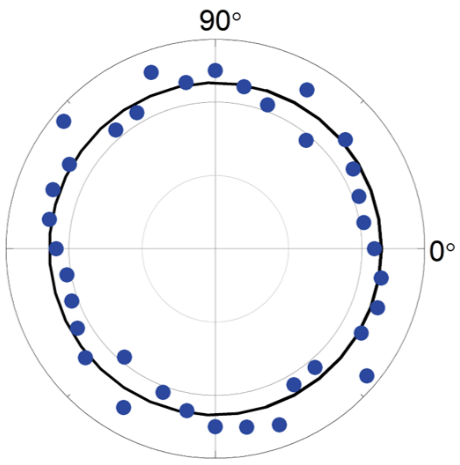

Fig. S12. TP-ASPL intensity versus excitation polarization angle (α) in polar coordinates. The collected polarization is fixed at some specific angle, the excitation polarization angle is rotated at 1270 nm. The intensity appears to be independent of the excitation polarization with trivial fluctuations in the polar plot.

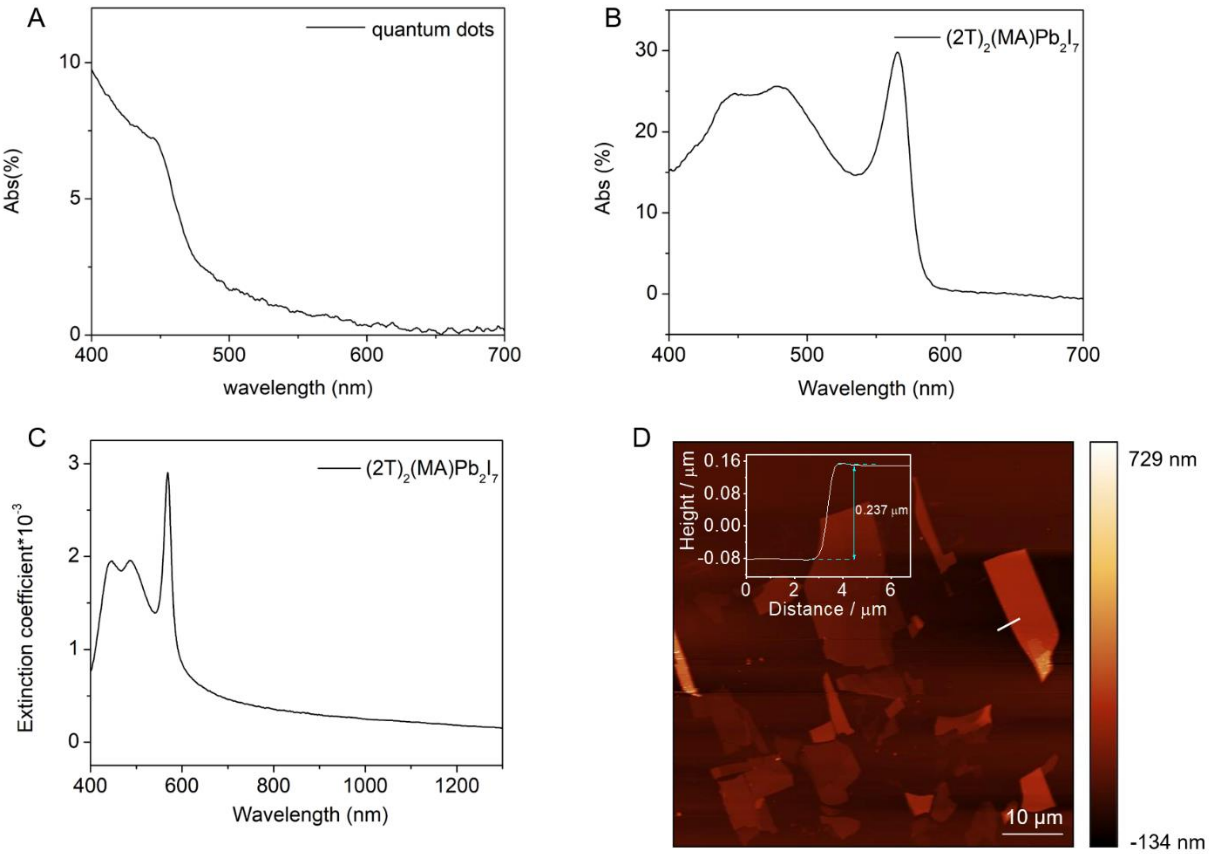

Fig. S13. Characterization of $(2T)_2(MA)Pb_2I_7$ and quantum dots for the analysis of PLQY. (**A**) Absorption spectrum of spin-coated quantum dots on a quartz substrate. (B) Micro-absorption spectrum of $(2T)_2(MA)Pb_2I_7$ with a thickness characterized by the atomic force microscopy (AFM) in (**D**). (**C**) Extinction coefficient of $(2T)_2(MA)Pb_2I_7$ for a full spectral range from 400 nm - 1300 nm according to Lambert Beer's law. (**D**) AFM image of $(2T)_2(MA)Pb_2I_7$, the thickness is determined to be ~ 237 nm. The absorption data can be used to calculate the photon ratios of PL to ASPL with Equations S5 and S6. The measured extinction coefficients can be used to derive the penetration depths with Equations S13 - S15.

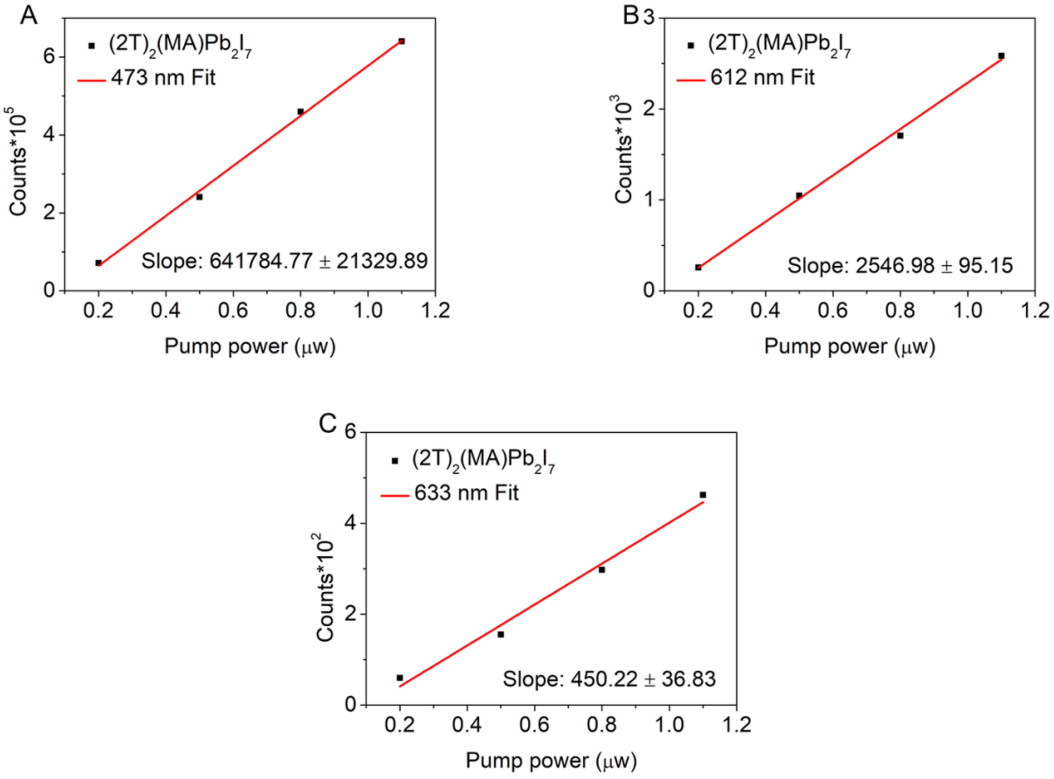

Fig. S14. Power-dependent PL for $(2T)_2(MA)Pb_2I_7$. The power-dependent PL excited at 473 nm (**A**), (**B**) 612 nm and 633 nm (**C**). All the power dependences indicate these testing powers are within the linear pump regime. The fitted slopes can be used to calculate the photon ratios of PL to ASPL with Equations S5 and S6.

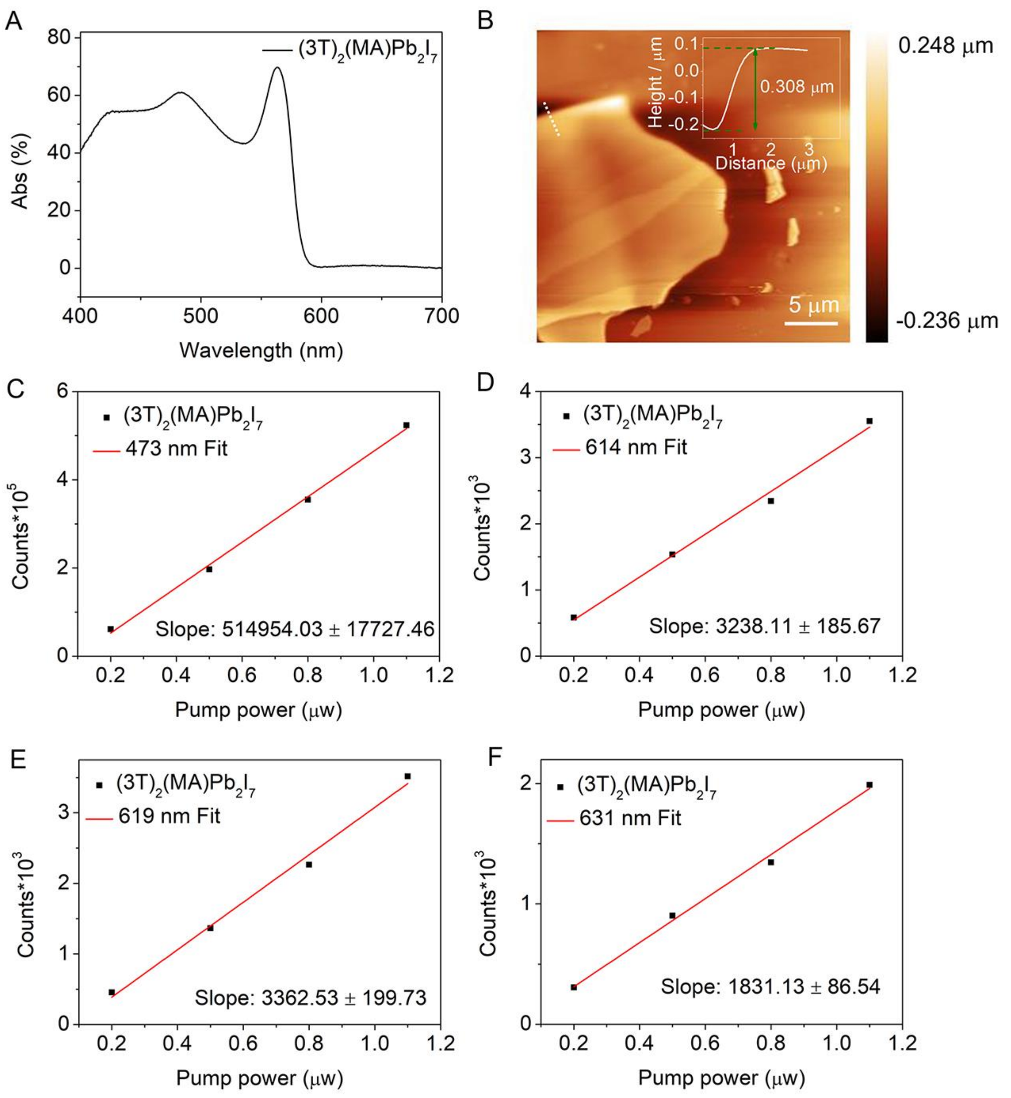

Fig. S15. Characterization of $(3T)_2(MA)Pb_2I_7$ and Power-dependent PL for the analysis of PLQY. (**A**) Micro-absorption spectrum of $(3T)_2(MA)Pb_2I_7$ with a thickness of ~ 308 nm characterized by the atomic force microscopy (AFM) in (**B**). The sample exhibited an absorption of 58.6% at 473 nm, while at 614 nm, 619 nm, and 631 nm, the absorption values were 0.7%, 0.8%, and 0.9% respectively. (**C**) The power-dependent PL excited at 473 nm, and the power-dependent ASPL at 614 nm (**D**), 619 nm (**E**) and 631 nm (**F**). All the power dependences indicate these testing powers are within the linear pump regime. The fitted slopes can be used to calculate the photon ratios of PL to ASPL with Equations S5 and S6.

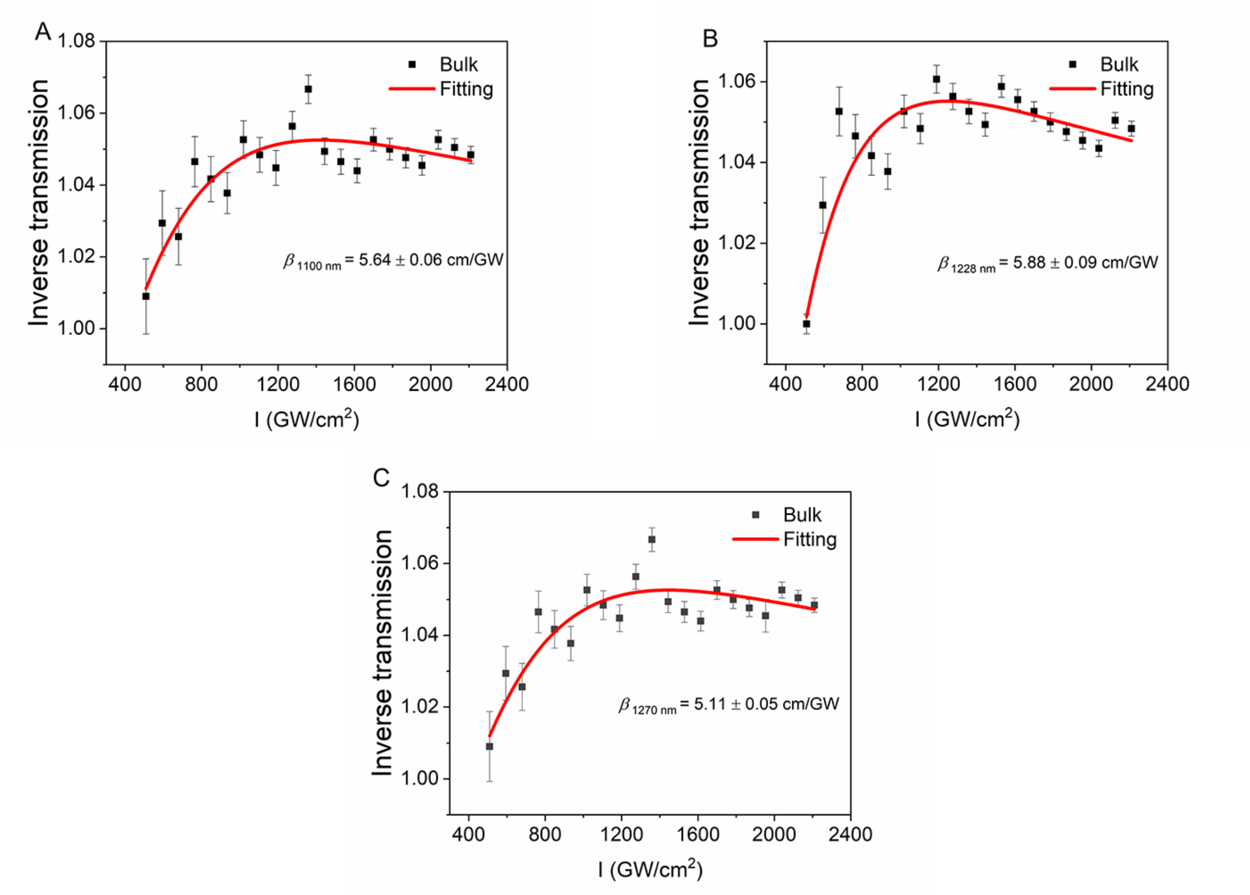

Fig. S16. Measurements of TPA coefficients under various pump conditions. According to Note S2, the TPA coefficients (β) can be extracted from pump-power dependent measurements with excitation wavelengths at 1100 nm (**A**), at 1220 nm (**B**) and at 1270 nm (**C**) as indicated in each sub-figure.

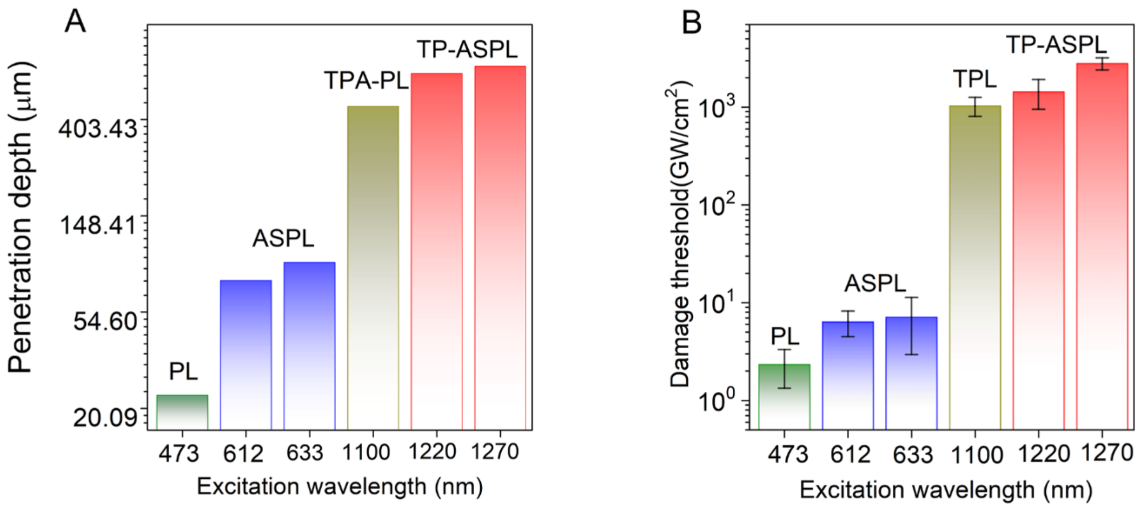

Fig. S17. Comparison of penetration depth and damage threshold under excitation of different wavelengths. (**A**) The measured damage penetration depths under 6 different cases according to Fig. S13 and Note S3. Similarly, TP-ASPL has the largest penetration depth, which gets close to the millimeter range. (**B**) The measured damage thresholds under 6 different cases, where the sample thickness is ~2 μm. It is apparent that TP-ASPL has the highest damage threshold (see Table S7).

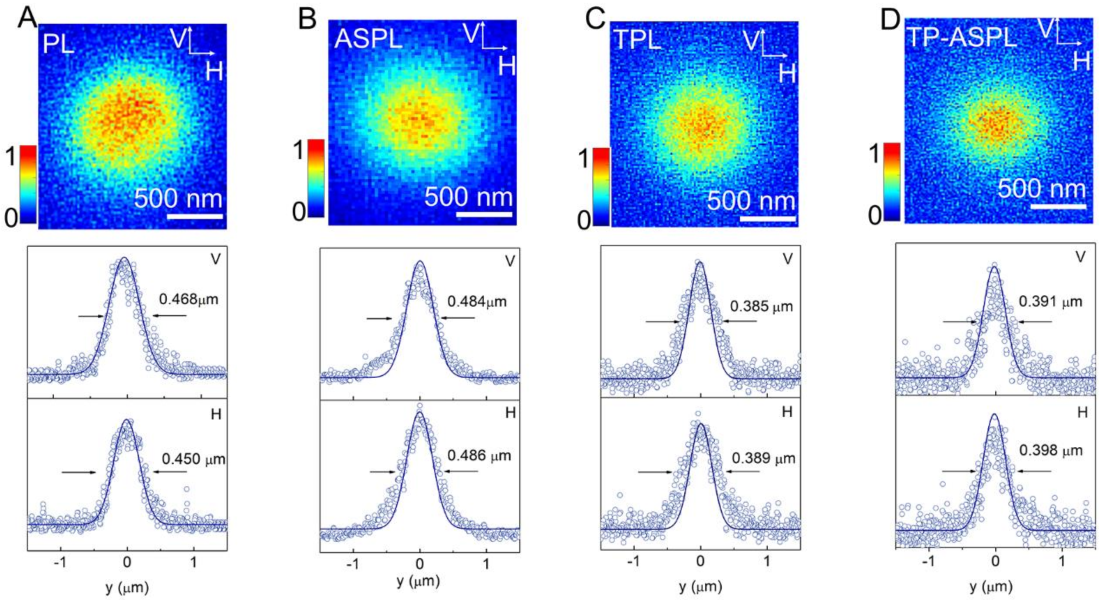

Fig. S18. The fluorescence imaging of point spread function (PSF). The PSF images of (**A**) PL excited at 473 nm, (**B**) ASPL excited at 633 nm, (**C**) two-photon absorption PL (TPA-PL) excited at 1100 nm, and (**D**) TP-ASPL excited at 1270 nm are collected in 2DPK of $(2T)_2(MA)Pb_2I_7$ with an objective of NA = 0.9. The images were collected by a home-built setup microscopy with single pixel of ~0.078 μm. Based on the Gaussian fitting of the spot size, it is obtained that the diameter of PSF for ASPL is around the diffraction limit while those of TPA-PL and TP-ASPL is below the diffraction limit.

Materials	UC energy gains (ΔE) with single-phonon absorption (meV)	References
Hexagonal boron nitride (hBN)	~165	(*65*)
Carbon nanotube	~ 120-130	(*7*)
Monolayer WSe_2	~ 31	(*5*)
CdSe/CdS QDs	~ 24	(*11*)
$(2T)_2(MA)Pb_2I_7$	~ 197	This work

Table S1. Summarized typical UC energy gains with single-phonon absorption in various materials.

	$(2T)_2MAPb_2I_7$
Crystal data	
Chemical formula	C21 H30 I7 N3 S4 Pb2
M_r	1755.40
Crystal system, space group	monoclinic, *Cm*
Temperature (K)	150.00
a, *b*, *c* (Å)	53.244(4), 6.1709(4), 6.1441(4)
α, β, γ (°)	90, 96.607(2), 90
V (Å^3)	2005.3(2)
Z	2
F (000)	1552
D_x (Mg m^{-3})	2.907
Radiation type	Mo *K*α
No. of reflections for cell measurement	9942
θ range (°) for cell measurement	2.312 to 27.64
μ (mm^{-1})	13.998
Crystal shape	Plate
Colour	Red
Crystal size (mm)	0.116 × 0.095 × 0.085
Data collection	
Diffractometer	Bruker AXS D8 Venture diffractometer with Photon III charge-integrating pixel array detector (CPAD)
Radiation source	High-intensity diamond-Cu/Mo hybrid dual-microfocal X-ray light source
Monochromator	Triumph curved graphite crystal
Photon III charge-integrating pixel array detector	10 × 14 cm^2
Scan method	w and phi scans
Absorption correction	Multi-scan *SADABS* 2016/2: Krause, L., Herbst-Irmer,

	R., Sheldrick G.M. & Stalke D. (2015). J. Appl. Cryst. 48, 3-10.
T_{min}, T_{max}	0.6238, 0.7456
No. of measured, independent and observed [$I > 2s(I)$] reflections	30782, 3729, 3456
R_{int}	0.0482
θ values (°)	2.310 to 24.712
$(\sin \theta/\lambda)_{max}$ (Å^{-1})	0.714
Range of h, k, l	h = -62 - 62, k = -7 - 7, l = -7 - 7
Refinement	
Refinement on	F^2
$R[F^2 > 2\sigma(F^2)]$, $wR(F^2)$, S	0.0429, 0.0895, 24.712
No. of reflections	3729
No. of parameters	330
No. of restraints	403
H-atom treatment	H-atom parameters constrained
Weighting scheme	$w = 1/[\sigma^2(F_o^2) + (0.0225P)^2 + 99.7832P]$ where $P = (F_o^2 + 2F_c^2)/3$
$(\Delta/\sigma)_{max}$	0.001
$\Delta\rho_{max}$, $\Delta\rho_{min}$ (e Å^{-3})	2.311 and -3.487
Absolute structure	0.223(18)

Table S2. Crystal data and structure refinement of $(2T)_2MAPb_2I_7$. The crystal structure of the material was refined using inversion twinning. The twin transformation matrix twin law (-1.0, 0.0, 0.0, 0.0, -1.0, 0.0, 0.0, 0.0, -1.0) was combined with inversion twinning, resulting in BASF values of 0.223(18). The disordered iodine atoms located around the lead ion are disordered in a 1:1 ratio around inversion centers. The atomic displacement parameters (ADPs) of the disordered Pb-I atoms were restrained to be similar using the ISOR 0.01 and DELU 0.01 commands of Shelxl. This disorder also causes 1:1 disorder of the ammonium ions that are hydrogen bonded to the iodine atoms, and the disorder extends into the bridging ethylene unit of the ligand. The two disordered $^{+}NH_3$-CH_2-CH_2- moieties were restrained to have similar geometries.

All of the 2T cations were restrained to have similar and rational geometries based on the previously established 2T cations model. The ADPs of the disordered C, N, and S atoms were restrained to be similar using the ISOR 0.01 and DELU 0.01 commands of Shelxl. The anisotropic temperature factors and Uij components of C, N, and S were restrained to be similar using the SIMU 0.01 command of Shelxl. The thiophene units were restrained to be flat due to the π-conjugation in the rigid 2T cations. The distances of C-N, C-S, and C-C were restrained to

be rationally consistent with organic fragments using the DFIX 0.01 command of Shelxl. Specifically, the C-N bond length was restrained to 1.55 Å, the π-conjugated C-C bond length was restrained to 1.4 Å, the non-π-conjugated C-C bond length was restrained to 1.55 Å, and the C-S bond length was restrained to 1.65 Å.

Excitation wavelength (nm)	PLQY or UCQY (%)
473	7.8
612	2.1
633	1.6

Table S3. PLQY and UCQY of $(2T)_2MAPb_2I_7$.

Excitation wavelength (nm)	PLQY or UCQY (%)
473	2.9
614	1.5
619	1.3
631	0.7

Table S4. PLQY and UCQY of $(3T)_2MAPb_2I_7$.

Material names	UCQY (ΔE with multi-phonon UC)	Reference
CdS nanoribbon	0.99 (up to ~ 110 meV)	(*3*)
$(PEA)_2PbI_4$	0.99 (~ 54 meV)	(*8*)
hBN	0.0056 (162 meV) (single-phonon UC)	(*65*)
$CsPbBr_3$	0.11 (158 meV)	(*66*)
$NH_4SCN:CsPbBr_3$	0.13 (153 meV)	
CdSe/CdS QDs	0.99 (48 meV)	(*11*)

Table S5. Summarized typical UCQY with different multi-phonon (single-phonon) UC gains in various materials.

Excitation wavelength (nm)	TPA coefficient β (cm/GW)
1100	5.64 ± 0.06
1228	5.88 ± 0.09
1270	5.11 ± 0.05

Table S6. TPA coefficients of $(2T)_2MAPb_2I_7$ in the nonlinear regime with different excitation wavelengths.

	473 nm	612 nm	633 nm	1100 nm	1228 nm	1270 nm
Damage threshold (GW/cm^2)	2.927928	9.684685	14.189189	1436.261261	1008.333333	3113.288288
	1.779279	5.180180	9.234234	1118.018018	1690.990991	3212.387387
	3.536036	5.405405	5.630631	820.720721	1238.288288	2759.459459
	2.702703	4.504505	2.927928	895.045045	985.360360	2830.180180
	0.698198	6.981982	3.603604	880.855856	2250.000000	2087.387387
Errors	0.991666	1.853974	4.161733	226.737670	480.139837	394.609316

Table S7. The measured damage thresholds under 6 different cases. The sample thickness ~2 μm.

	473 nm	612 nm	633 nm	1100 nm	1228 nm	1270 nm
Damage threshold (GW/cm^2)	0.008484	0.402999	0.636314	339.367655	127.262870	636.314352
	0.169684	0.445420	0.381789	254.525741	190.894306	424.209568
	0.148473	0.360578	0.678735	296.946698	636.314352	1060.523921
	0.190894	0.330883	0.445420	1060.523921	1060.523921	763.577223
	0.309673	0.424210	0.445420	636.314352	848.419136	296.946698
Errors	0.096325	0.041788	0.117407	302.827401	363.931265	266.948849

Table S8. The measured damage thresholds under 6 different cases. The sample thickness is around ~35 nm.

Materials	Laser (wavelength / pulse width)	Damage threshold (peak power in GW/cm^2)	References
Monolayer / few-layer MoS_2	1030 nm, 340 fs	~70-97	(*63*)
1-3L WS_2	1030 nm, 340 fs	~68	(*67*)
$(PEA)_2PbI_4$	800 nm, 100 fs	0.9	(*16*)
R-$(MPEA)_{1.5}$ $PbBr_{3.5}$ $(DMSO)_{0.5}$	720-980 nm, 100 fs	5.2	(*68*)
Polymethyl methacrylate (PMMA) nanofibers	532 nm, 5ns	0.1-0.2	(*69*)
$(2T)_2(MA)Pb_2I_7$	633 nm; 220 fs<br>1270 nm; 220 fs	$0.5\pm0.1 - 7.1\pm4.2$<br>$(6.4\pm2.7)\times10^2 - (2.8\pm0.4)\times10^3$	This work

Table S9. Summarized typical damage threshold of nonlinear UC materials. The damage threshold ranges of $(2T)_2(MA)Pb_2I_7$ are based on statistical analysis of samples with thickness of ~35 nm and ~2 μm.

REFERENCES AND NOTES

1. P. Pringsheim, Zwei bemerkungen über den unterschied von lumineszenz- und temperaturstrahlung. *Z. Phys.* **57**, 739–746 (1929).

2. R. I. Epstein, M. I. Buchwald, B. C. Edwards, T. R. Gosnell, C. E. Mungan, Observation of laser-induced fluorescent cooling of a solid. *Nature* **377**, 500–503 (1995).

3. J. Zhang, D. Li, R. Chen, Q. Xiong, Laser cooling of a semiconductor by 40 kelvin. *Nature* **493**, 504–508 (2013).

4. Y. Gao, J.-M. Lai, J. Zhang, Phonon-assisted upconversion photoluminescence of quantum emitters. *J. Semicond.* **44**, 041901 (2023).

5. A. M. Jones, H. Yu, J. R. Schaibley, J. Yan, D. G. Mandrus, T. Taniguchi, K. Watanabe, H. Dery, W. Yao, X. Xu, Excitonic luminescence upconversion in a two-dimensional semiconductor. *Nat. Phys.* **12**, 323–327 (2016).

6. B. Wu, A. Wang, J. Fu, Y. Zhang, C. Yang, Y. Gong, C. Jiang, M. Long, G. Zhou, S. Yue, W. Ma, X. Liu, Uncovering the mechanisms of efficient upconversion in two-dimensional perovskites with anti-Stokes shift up to 220 meV. *Sci. Adv.* **9**, eadi9347 (2023).

7. N. Akizuki, S. Aota, S. Mouri, K. Matsuda, Y. Miyauchi, Efficient near-infrared up-conversion photoluminescence in carbon nanotubes. *Nat. Commun.* **6**, 8920 (2015).

8. S.-T. Ha, C. Shen, J. Zhang, Q. Xiong, Laser cooling of organic–inorganic lead halide perovskites. *Nat. Photonics* **10**, 115–121 (2016).

9. M. Manca, M. M. Glazov, C. Robert, F. Cadiz, T. Taniguchi, K. Watanabe, E. Courtade, T. Amand, P. Renucci, X. Marie, G. Wang, B. Urbaszek, Enabling valley selective exciton scattering in monolayer WSe_2 through upconversion. *Nat. Commun.* **8**, 14927 (2017).

10. J. Jadczak, L. Bryja, J. Kutrowska-Girzycka, P. Kapuscinski, M. Bieniek, Y. S. Huang, P. Hawrylak, Room temperature multi-phonon upconversion photoluminescence in monolayer semiconductor WS_2. *Nat. Commun.* **10**, 107 (2019).

11. Z. Ye, X. Lin, N. Wang, J. Zhou, M. Zhu, H. Qin, X. Peng, Phonon-assisted up-conversion photoluminescence of quantum dots. *Nat. Commun.* **12**, 4283 (2021).

12. J. M. Lai, Y. J. Sun, Q. H. Tan, P. H. Tan, J. Zhang, Laser cooling of a lattice vibration in van der waals semiconductor. *Nano Lett.* **22**, 7129–7135 (2022).

13. D. V. Seletskiy, S. D. Melgaard, S. Bigotta, A. Di Lieto, M. Tonelli, M. Sheik-Bahae, Laser cooling of solids to cryogenic temperatures. *Nat. Photonics* **4**, 161–164 (2010).

14. F. Wang, R. Deng, J. Wang, Q. Wang, Y. Han, H. Zhu, X. Chen, X. Liu, Tuning upconversion through energy migration in core-shell nanoparticles. *Nat. Mater.* **10**, 968–973 (2011).

15. Z. Hu, V. S. Khadka, W. Wang, D. W. Galipeau, X. Yan, Theoretical study of two-photon absorption properties and up-conversion efficiency of new symmetric organic π-conjugated molecules for photovoltaic devices. *J. Mol. Model.* **18**, 3657–3667 (2012).

16. W. Liu, J. Xing, J. Zhao, X. Wen, K. Wang, P. Lu, Q. Xiong, Giant two-photon absorption and its saturation in 2D organic–inorganic perovskite. *Adv Opt Mater* **5**, 1601045 (2017).

17. Y. Liu, Y. Lu, X. Yang, X. Zheng, S. Wen, F. Wang, X. Vidal, J. Zhao, D. Liu, Z. Zhou, C. Ma, J. Zhou, J. A. Piper, P. Xi, D. Jin, Amplified stimulated emission in upconversion nanoparticles for super-resolution nanoscopy. *Nature* **543**, 229–233 (2017).

18. A. Fernandez-Bravo, K. Yao, E. S. Barnard, N. J. Borys, E. S. Levy, B. Tian, C. A. Tajon, L. Moretti, M. V. Altoe, S. Aloni, K. Beketayev, F. Scotognella, B. E. Cohen, E. M. Chan, P. J. Schuck, Continuous-wave upconverting nanoparticle microlasers. *Nat. Nanotechnol.* **13**, 572–577 (2018).

19. D. J. Garfield, N. J. Borys, S. M. Hamed, N. A. Torquato, C. A. Tajon, B. Tian, B. Shevitski, E. S. Barnard, Y. D. Suh, S. Aloni, J. B. Neaton, E. M. Chan, B. E. Cohen, P. J. Schuck, Enrichment of molecular antenna triplets amplifies upconverting nanoparticle emission. *Nat. Photonics* **12**, 402–407 (2018).

20. B. D. Ravetz, A. B. Pun, E. M. Churchill, D. N. Congreve, T. Rovis, L. M. Campos, Photoredox catalysis using infrared light via triplet fusion upconversion. *Nature* **565**, 343–346 (2019).

21. S. Wen, J. Zhou, P. J. Schuck, Y. D. Suh, T. W. Schmidt, D. Jin, Future and challenges for hybrid upconversion nanosystems. *Nat. Photonics* **13**, 828–838 (2019).

22. C. Lee, E. Z. Xu, Y. Liu, A. Teitelboim, K. Yao, A. Fernandez-Bravo, A. M. Kotulska, S. H. Nam, Y. D. Suh, A. Bednarkiewicz, B. E. Cohen, E. M. Chan, P. J. Schuck, Giant nonlinear optical responses from photon-avalanching nanoparticles. *Nature* **589**, 230–235 (2021).

23. B. S. Richards, D. Hudry, D. Busko, A. Turshatov, I. A. Howard, Photon upconversion for photovoltaics and photocatalysis: A critical review. *Chem. Rev.* **121**, 9165–9195 (2021).

24. H. Chen, Z. Jiang, H. Hu, B. Kang, B. Zhang, X. Mi, L. Guo, C. Zhang, J. Li, J. Lu, L. Yan, Z. Fu, Z. Zhang, H. Zheng, H. Xu, Sub-50-ns ultrafast upconversion luminescence of a rare-earth-doped nanoparticle. *Nat. Photonics* **16**, 651–657 (2022).

25. H. Hong, C. Wu, Z. Zhao, Y. Zuo, J. Wang, C. Liu, J. Zhang, F. Wang, J. Feng, H. Shen, J. Yin, Y. Wu, Y. Zhao, K. Liu, P. Gao, S. Meng, S. Wu, Z. Sun, K. Liu, J. Xiong, Giant enhancement of optical nonlinearity in two-dimensional materials by multiphoton-excitation resonance energy transfer from quantum dots. *Nat. Photonics* **15**, 510–515 (2021).

26. F. Auzel, Upconversion and anti-Stokes processes with f and d ions in solids. *Chem. Rev.* **104**, 139–174 (2004).

27. J. F. Suyver, A. Aebischer, D. Biner, P. Gerner, J. Grimm, S. Heer, K. W. Krämer, C. Reinhard, H. U. Güdel, Novel materials doped with trivalent lanthanides and transition metal ions showing near-infrared to visible photon upconversion. *Opt. Mater.* **27**, 1111–1130 (2005).

28. X. Zheng, Z. Li, Y. Zhang, M. Chen, T. Liu, C. Xiao, D. Gao, J. B. Patel, D. Kuciauskas, A. Magomedov, R. A. Scheidt, X. Wang, S. P. Harvey, Z. Dai, C. Zhang, D. Morales, H. Pruett, B. M. Wieliczka, A. R. Kirmani, N. P. Padture, K. R. Graham, Y. Yan, M. K. Nazeeruddin, M.

D. McGehee, Z. Zhu, J. M. Luther, Co-deposition of hole-selective contact and absorber for improving the processability of perovskite solar cells. *Nat. Energy* **8**, 462–472 (2023).

29. W. Liang, C. Nie, J. Du, Y. Han, G. Zhao, F. Yang, G. Liang, K. Wu, Near-infrared photon upconversion and solar synthesis using lead-free nanocrystals. *Nat. Photonics* **17**, 346–353 (2023).

30. Y. Wu, J. Xu, E. T. Poh, L. Liang, H. Liu, J. K. W. Yang, C.-W. Qiu, R. A. L. Vallée, X. Liu, Upconversion superburst with sub-2 μs lifetime. *Nat. Nanotechnol.* **14**, 1110–1115 (2019).

31. R. Chikkaraddy, R. Arul, L. A. Jakob, J. J. Baumberg, Single-molecule mid-infrared spectroscopy and detection through vibrationally assisted luminescence. *Nat. Photonics* **17**, 865–871 (2023).

32. L. Xiong, Z. Chen, Q. Tian, T. Cao, C. Xu, F. Li, High contrast upconversion luminescence targeted imaging in vivo using peptide-labeled nanophosphors. *Anal. Chem.* **81**, 8687–8694 (2009).

33. A. Xomalis, X. Zheng, R. Chikkaraddy, Z. Koczor-Benda, E. Miele, E. Rosta, G. A. E. Vandenbosch, A. Martínez, J. J. Baumberg, Detecting mid-infrared light by molecular frequency upconversion in dual-wavelength nanoantennas. *Science* **374**, 1268–1271 (2021).

34. W. Chen, P. Roelli, H. Hu, S. Verlekar, S. P. Amirtharaj, A. I. Barreda, T. J. Kippenberg, M. Kovylina, E. Verhagen, A. Martínez, C. Galland, Continuous-wave frequency upconversion with a molecular optomechanical nanocavity. *Science* **374**, 1264–1267 (2021).

35. S.-Q. Hu, H. Zhao, X.-B. Liu, Q. Chen, D.-Q. Chen, X.-Y. Zhang, S. Meng, Phonon-coupled high-harmonic generation for exploring nonadiabatic electron-phonon interactions. *Phys. Rev. Lett.* **133**, 156901 (2024).

36. D. Golež, Z. Sun, Y. Murakami, A. Georges, A. J. Millis, Nonlinear spectroscopy of collective modes in an excitonic insulator. *Phys. Rev. Lett.* **125**, 257601 (2020).

37. Y. Yamada, Y. Kanemitsu, Electron-phonon interactions in halide perovskites. *NPG Asia Mater.* **14**, 48 (2022).

38. A. R. Srimath Kandada, H. Li, E. R. Bittner, C. Silva-Acuña, Homogeneous optical line widths in hybrid ruddlesden–popper metal halides can only be measured using nonlinear spectroscopy. *J. Phys. Chem. C* **126**, 5378–5387 (2022).

39. Q. Liu, Y. Zhang, C. S. Peng, T. Yang, L. M. Joubert, S. Chu, Single upconversion nanoparticle imaging at sub-10 W cm^{-2} irradiance. *Nat. Photonics* **12**, 548–553 (2018).

40. J. C. Blancon, A. V. Stier, H. Tsai, W. Nie, C. C. Stoumpos, B. Traore, L. Pedesseau, M. Kepenekian, F. Katsutani, G. T. Noe, J. Kono, S. Tretiak, S. A. Crooker, C. Katan, M. G. Kanatzidis, J. J. Crochet, J. Even, A. D. Mohite, Scaling law for excitons in 2D perovskite quantum wells. *Nat. Commun.* **9**, 2254 (2018).

41. J. C. Blancon, J. Even, C. C. Stoumpos, M. G. Kanatzidis, A. D. Mohite, Semiconductor physics of organic-inorganic 2D halide perovskites. *Nat. Nanotechnol.* **15**, 969–985 (2020).

42. J. Y. Park, R. Song, J. Liang, L. Jin, K. Wang, S. Li, E. Shi, Y. Gao, M. Zeller, S. J. Teat, P. Guo, L. Huang, Y. S. Zhao, V. Blum, L. Dou, Thickness control of organic semiconductor-incorporated perovskites. *Nat. Chem.* **15**, 1745–1753 (2023).

43. J. L. Clark, G. Rumbles, Laser cooling in the condensed phase by frequency up-conversion. *Phys. Rev. Lett.* **76**, 2037–2040 (1996).

44. M. Li, P. Huang, H. Zhong, Current understanding of band edge properties of halide perovskites urbach tail rashba splitting and exciton binding energy. *J. Phys. Chem. Lett.* **14**, 1592–1603 (2023).

45. J. Lu, Z. Gan, J. van Embden, B. Jia, X. Wen, Photon upconversion in metal halide perovskites. *Mater. Today* **86**, 340–355 (2025).

46. W. Zhang, Y. Ye, C. Liu, J. Wang, J. Ruan, X. Zhao, J. Han, Two-step anti-Stokes photoluminescence of $CsPbX_3$ nanocrystals. *Adv Opt Mater* **9**, 2001885 (2021).

47. E. Shi, B. Yuan, S. B. Shiring, Y. Gao, Akriti, Y. Guo, C. Su, M. Lai, P. Yang, J. Kong, B. M. Savoie, Y. Yu, L. Dou, Two-dimensional halide perovskite lateral epitaxial heterostructures. *Nature* **580**, 614–620 (2020).

48. S. Kilina, D. Kilin, S. Tretiak, Light-driven and phonon-assisted dynamics in organic and semiconductor nanostructures. *Chem. Rev.* **115**, 5929–5978 (2015).

49. G. Cohen, J. B. Haber, J. B. Neaton, D. Y. Qiu, S. Refaely-Abramson, Phonon-driven femtosecond dynamics of excitons in crystalline pentacene from first principles. *Phys. Rev. Lett.* **132**, 126902 (2024).

50. L. P. René de Cotret, J.-H. Pöhls, M. J. Stern, M. R. Otto, M. Sutton, B. J. Siwick, Time- and momentum-resolved phonon population dynamics with ultrafast electron diffuse scattering. *Phys. Rev. B* **100**, 214115 (2019).

51. J. J. Geuchies, J. Klarbring, L. D. Virgilio, S. Fu, S. Qu, G. Liu, H. Wang, J. M. Frost, A. Walsh, M. Bonn, H. Kim, Anisotropic electron–phonon interactions in 2D lead-halide perovskites. *Nano Lett.* **24**, 8642–8649 (2024).

52. D. B. Straus, S. Hurtado Parra, N. Iotov, J. Gebhardt, A. M. Rappe, J. E. Subotnik, J. M. Kikkawa, C. R. Kagan, Direct observation of electron–phonon coupling and slow vibrational relaxation in organic–inorganic hybrid perovskites. *J. Am. Chem. Soc.* **138**, 13798–13801 (2016).

53. M. Schlipf, S. Poncé, F. Giustino, Carrier lifetimes and polaronic mass enhancement in the hybrid halide perovskite $CH_3NH_3PbI_3$ from multiphonon Fröhlich coupling. *Phys. Rev. Lett.* **121**, 086402 (2018).

54. M. Guan, D. Chen, Q. Chen, Y. Yao, S. Meng, Coherent phonon assisted ultrafast order-parameter reversal and hidden metallic state in Ta_2NiSe_5. *Phys. Rev. Lett.* **131**, 256503 (2023).

55. Y. Zhang, W. Du, X. Liu, Photophysics and its application in photon upconversion. *Nanoscale* **16**, 2747–2764 (2024).

56. T. Reiker, Z. Liu, C. Winter, M. V. Cappellari, D. G. Abradelo, C. A. Strassert, D. Zhang, H. Zacharias, Ultrafast electron dynamics in excited states of conjugated thiophene–fluorene organic polymer (pF8T2) thin films. *Phys. Chem. Chem. Phys.* **26**, 4736–4751 (2024).

57. E. Rueda, J. Serna, J. I. Uribe, D. Ramírez, F. Jaramillo, J. Osorio, H. García, Nonlinear two-photon absorption in the near-infrared band for lead bromide perovskite films using an F-scan nonlinear spectrometer. *ACS Omega* **7**, 29100–29105 (2022).

58. M. J. Frisch, G. W. Trucks, H. B. Schlegel, G. E. Scuseria, M. A. Robb, J. R. Cheeseman, G. Scalmani, V. Barone, G. A. Petersson, H. Nakatsuji, M. C. X. Li, A. V. Marenich, J. Bloino, B. G. Janesko, R. Gomperts, B. Mennucci, H. P. Hratchian, J. V. Ortiz, A. F. Izmaylov, J. L. Sonnenberg, D. Williams-Young, F. Ding, F. Lipparini, F. Egidi, J. Goings, B. Peng, A. Petrone, T. Henderson, D. Ranasinghe, V. G. Zakrzewski, J. Gao, N. Rega, G. Zheng, W. Liang, M. Hada, M. Ehara, K. Toyota, R. Fukuda, J. Hasegawa, M. Ishida, T. Nakajima, Y. Honda, O. Kitao, H. Nakai, T. Vreven, K. Throssell, J. A. Montgomery, Jr., J. E. Peralta, F. Ogliaro, M. J. Bearpark, J. J. Heyd, E. N. Brothers, K. N. Kudin, V. N. Staroverov, T. A. Keith, R. Kobayashi, J. Normand, K. Raghavachari, A. P. Rendell, J. C. Burant, S. S. Iyengar, J. Tomasi, M. Cossi, J. M. Millam, M. Klene, C. Adamo, R. Cammi, J. W. Ochterski, R. L. Martin, K. Morokuma, O. Farkas, J. B. Foresman, D. J. Fox, Gaussian 16 (Gaussian Inc., 2016).

59. R. W. Boyd, *Nonlinear Optics* (Springer, 2008).

60. M. Sheik-Bahae, A. A. Said, T.-H. Wei, D. J. Hagan, E. W. V. Stryland, Sensitive measurement of optical nonlinearities using a single beam. *IEEE J. Quantum Electron.* **26**, 760–769 (1990).

61. X. Dai, X. Zhang, I. M. Kislyakov, L. Wang, J. Huang, S. Zhang, N. Dong, J. Wang, Enhanced two-photon absorption and two-photon luminescence in monolayer MoS_2 and WS_2 by defect repairing. *Opt. Express* **27**, 13744–13753 (2019).

62. N. Dong, Y. Li, S. Zhang, N. McEvoy, R. Gatensby, G. S. Duesberg, J. Wang, Saturation of two-photon absorption in layered transition metal dichalcogenides: Experiment and theory. *ACS Photonics* **5**, 1558–1565 (2018).

63. Y. Li, N. Dong, S. Zhang, X. Zhang, Y. Feng, K. Wang, L. Zhang, J. Wang, Giant two-photon absorption in monolayer MoS_2. *Laser Photonics Rev.* **9**, 427–434 (2015).

64. Z. Zhang, Z. Xu, Y. Song, T. Liu, B. Dong, J. Liu, H. Wang, Interfacial stress characterization of GaN epitaxial layer with sapphire substrate by confocal Raman spectroscopy. *Nanotechnol. Precis. Eng.* **4**, 023002 (2021).

65. Q. Wang, Q. Zhang, X. Zhao, X. Luo, C. P. Y. Wong, J. Wang, D. Wan, T. Venkatesan, S. J. Pennycook, K. P. Loh, G. Eda, A. T. S. Wee, Photoluminescence upconversion by defects in hexagonal boron nitride. *Nano Lett.* **18**, 6898–6905 (2018).

66. B. J. Roman, M. Sheldon, The role of mid-gap states in all-inorganic $CsPbBr_3$ nanoparticle one photon up-conversion. *Chem. Commun.* **54**, 6851–6854 (2018).

67. S. Zhang, N. Dong, N. McEvoy, M. O'Brien, S. Winters, N. C. Berner, C. Yim, Y. Li, X. Zhang, Z. Chen, L. Zhang, G. S. Duesberg, J. Wang, Direct observation of degenerate two-photon absorption and its saturation in WS_2 and MoS_2 monolayer and few-layer films. *ACS Nano* **9**, 7142–7150 (2015).

68. C. Yuan, X. Li, S. Semin, Y. Feng, T. Rasing, J. Xu, Chiral lead halide perovskite nanowires for second-order nonlinear optics. *Nano Lett.* **18**, 5411–5417 (2018).

69. C. Yogeswari, K. M. Hijas, M. Abith, T. C. Sabari Girisun, R. Nagalakshmi, Intensity-dependent two-photon absorption and its saturation in 2-methyl 4-nitroaniline nanofibers. *J. Mater. Sci. Mater. Electron.* **32**, 360–372 (2021).
